# Supplementary material for: Adaptable graphitic C6N6-based copper single-atom catalyst for intelligent biosensing
Source: Nat Commun. 2023 May 15;14:2780. doi: 10.1038/s41467-023-38459-9 (PMC10185664; doi:10.1038/s41467-023-38459-9)
Supplement: Supplementary file 1 — Supplementary Information [file 41467_2023_38459_MOESM1_ESM.pdf]

Supplementary Information

Adaptable Graphitic C_6N_6-Based Copper Single-Atom Catalyst for Intelligent Biosensing

Qing Hong, ‡[1] Hong Yang, ‡[1] Yanfeng Fang,[1] Wang Li,[1] Caixia Zhu,[1] Zhuang Wang,[1] Sicheng Liang,[1] Xuwen Cao,[1] Zhixin Zhou,[1] Yanfei Shen*[2], Songqin Liu,[1] Yuanjian Zhang*[1]

[1]Jiangsu Engineering Laboratory of Smart Carbon-Rich Materials and Device, Jiangsu Province Hi-Tech Key Laboratory for Bio-Medical Research, School of Chemistry and Chemical Engineering, Southeast University, Nanjing 211189, China, Email: Yuanjian.Zhang@seu.edu.cn

[2]Medical School, Southeast University, Nanjing 210009, China, Email: Yanfei.Shen@seu.edu.cn

‡ These authors contributed equally.

Table of Contents

Methods. 4

Supplementary Fig. 1. XRD patterns of Cu-$CN_{int.}$ prepared from Cu/DCDA complex and mixture. 10

Supplementary Fig. 2. High-resolution C 1*s* XPS spectra of PCN and CN_{mw}. 11

Supplementary Fig. 3. Matrix-free LDI-TOF mass spectrum of $Cu_{SA}C_6N_6$. 12

Supplementary Fig. 4. SEM images of $Cu_{SA}C_6N_6$, PCN and CN_{mw}. 13

Supplementary Fig. 5. High-resolution TEM images of the $Cu_{SA}C_6N_6$ with different magnifications. 14

Supplementary Fig. 6. HAADF-STEM images of the $Cu_{SA}C_6N_6$ in other area. 15

Supplementary Fig. 7. TEM image and corresponding STEM-EDS elemental mapping images of $Cu_{SA}C_6N_6$. 16

Supplementary Fig. 8. TEM image and corresponding STEM-EDS elemental mapping images of $Cu_{SA}C_6N_6$ in other area. 17

Supplementary Fig. 9. High-resolution N 1*s* XPS spectra of $Cu_{SA}C_6N_6$. 18

Supplementary Fig. 10. High-resolution Cu 2*p* XPS spectra of $Cu_{SA}C_6N_6$. 19

Supplementary Fig. 11. UV-vis absorption spectra of ABTS and that catalyzed by $Cu_{SA}C_6N_6$. 20

Supplementary Fig. 12. Ultraviolet-visible-near-infrared absorption spectrum of $Cu_{SA}C_6N_6$. 21

Supplementary Fig. 13. Kubelka-Munk plot of $Cu_{SA}C_6N_6$ powders. 22

Supplementary Fig. 14. Emission spectrum of white household LED lamp. 23

Supplementary Fig. 15. Peroxidase-like velocity of $Cu_{SA}C_6N_6$ in basic and gain reaction. 24

Supplementary Fig. 16. Ultraviolet-visible-near-infrared absorption spectrum of CN_{mw}. 25

Supplementary Fig. 17. Kubelka-Munk plot of CN_{mw} powders. 26

Supplementary Fig. 18. Nyquist plots of $Cu_{SA}C_6N_6$, Cu-N-C/CN_{mw} and PCN. 27

Supplementary Fig. 19. PL spectra of $Cu_{SA}C_6N_6$, Cu-N-C/CN_{mw} and PCN. 28

Supplementary Fig. 20. PEC experiments of $Cu_{SA}C_6N_6$, Cu-N-C/CN_{mw} and PCN. 29

Supplementary Fig. 21. Peroxidase-like velocity of $Cu_{SA}C_6N_6$ in basic reaction. 30

Supplementary Fig. 22. Thermal images showing the photothermal effect of $Cu_{SA}C_6N_6$. 31

Supplementary Fig. 23. Thermal images of $Cu_{SA}C_6N_6$. 32

Supplementary Fig. 24. Absorbance of $ABTS_{ox}$ catalyzed by $Cu_{SA}C_6N_6$ under same temperature. 33

Supplementary Fig. 25. Absorbance of $ABTS_{ox}$ with control experiments. 34

Supplementary Fig. 26. Absorbance of $ABTS_{ox}$ catalyzed by $Cu_{SA}C_6N_6$ with various Cu contents. 35

Supplementary Fig. 27. Absorbance of $ABTS_{ox}$ catalyzed by $Cu_{SA}C_6N_6$ with tuning the irradiation power density. 36

Supplementary Fig. 28. Peroxidase-like activity of $Cu_{SA}C_6N_6$ with different scavengers in basic reaction. 37

Supplementary Fig. 29. Peroxidase-like activity of $Cu_{SA}C_6N_6$ with different scavengers in gain reaction. 38

Supplementary Fig. 30. UV absorption spectra of NBT_{re} catalyzed by $Cu_{SA}C_6N_6$. 39

Supplementary Fig. 31. PL spectra of umbelliferone catalyzed by $Cu_{SA}C_6N_6$. 40

Supplementary Fig. 32. Simulated absorption spectra of $Cu_{SA}C_6N_6$, Cu-g-C_3N_4, and Cu-PCN.41

Supplementary Fig. 33. Isosurfaces of hole and electron distribution for $Cu_{SA}C_6N_6$, Cu-g-C_3N_4, and Cu-PCN. 42

Supplementary Fig. 34. Dominant contributions of molecular orbital transitions for $Cu_{SA}C_6N_6$.43

Supplementary Fig. 35. Dominant contributions of molecular orbital transitions for Cu-g-C_3N_4. 44

Supplementary Fig. 36. Dominant contributions of molecular orbital transitions for Cu-PCN. 45

Supplementary Fig. 37. Partition and CTS of C_6N_6. 46

Supplementary Fig. 38. Partition and CTS of three different $Cu_{SA}C_6N_6$. 47

Supplementary Fig. 39. Energy curve of optimization and corresponding CTS of $Cu_{SA}C_6N_6$. 48

Supplementary Fig. 40. CTS of $Cu_{SA}C_6N_6$ calculated by PBE0 and ωB97XD. 49

Supplementary Table 1. Combustion elemental analysis of $Cu_{SA}C_6N_6$ and CN_{mw}. 50

Supplementary Table 2. EXAFS fitting parameters at the Cu K-edge for various samples. 51

Kinetic equation of glucose sensor under light irradiation of different intensity. 52

References. 58

Methods

Reagent. Dicyandiamide (DCDA, 99%), ethylene glycol (EG), 2,2'-azinobis-(3-ethylbenzthiazoline-6-sulphonate) (ABTS), superoxide dismutase (SOD, from bovine erythrocytes, ≥3,000 units/mg protein), 5,5-dimethyl-1-pyrroline N-oxide (DMPO) and 2,2,6,6-tetramethylpiperidine (TEMP) were purchased from Sigma-Aldrich; Copper (II) acetate monohydrate ($Cu(CH_3COO)_2{\cdot}H_2O$), copper (II) chloride dihydrate ($CuCl_2{\cdot}2H_2O$), copper (II) sulfate pentahydrate ($CuSO_4{\cdot}5H_2O$), acetic acid (HAc), sodium acetate trihydrate ($NaAc{\cdot}3H_2O$) and ethanol were purchased from Sinopharm Chemical Reagent Co., Ltd. (China). Isopropanol (IPA, 99.8%,), ethylenediaminetetraacetic acid disodium salt (EDTA-Na, 99%), glucose, glucose oxidase from aspergillus niger (GO_x) and hydrogen peroxide (H_2O_2, 30%) were purchased from Aladdin Chemistry Co., Ltd. (China). All chemicals were used without further purification, unless otherwise specified. Ultrapure water (18.2 MΩ cm) used in all the experiments was obtained from a Direct-Q 3 UV pure water purification system (Millipore, USA).

Synthesis of polymeric carbon nitride (PCN). PCN was prepared according to the previously reported literatures.[1] Briefly, 10 g of DCDA was placed in a 30 mL capped crucible and heated at 550 °C in air and kept at this temperature for 4 h in a muffle furnace. The obtained yellow product, i.e. PCN, was ground into fine powder and used without further purification.

Synthesis of Cu^{2+}-PCN. Cu^{2+}-PCN was prepared according to the previously reported literatures.[2] Firstly, 1 g of as-prepared PCN was placed in a ball mill and added into 2 mL of pure water to prepare the PCN nanosheets at 3000 rpm for 4 h. Then, 5 mL of PCN nanosheets aqueous solution (1 mg/mL) and 30 mg of $Cu(CH_3COO)_2{\cdot}H_2O$ (0.15 mmol) were under ultrasonic for 30 min. After ultrasonic treatment, the solution was centrifuged and washed with ultrapure water for several times to remove the unbounded Cu^{2+}. Finally, the product was dried in a vacuum oven overnight.

Synthesis of Cu-N-C SAzymes. Cu-N-C SAzymes was prepared according to the

previously reported literatures.[3] Briefly, 30 mg $Cu(CH_3COO)_2·H_2O$ and 500 g of KCl acted as the template were stirred with a 30 mL of absolute methanol, and dried at 80 °C for immobilized Cu^{2+} ions onto the surface of KCl. Next, the 30 mL of methanol solution including 0.8 g of 2-MeIm was added for immobilization of the 2-MeIm onto the KCl template. And the solution was dried at 80 °C to obtain Cu(2-MeIm)/KCl. Then, the resulting powder was heated at a rate of 10 °C min^{-1} to reach 750 °C and kept at this temperature for 2 h under Ar flow. Finally, the bulk sample was washed with diluted H_2SO_4 (0.5 M) and H_2O several times.

Synthesis of $Cu_{SA}C_6N_6$ and CN_{mw}. Briefly, 20 g of DCDA precursor was added into 200 mL of EG under stirring at 80 °C for 30 min to form a true solution. Then, 15 mg $Cu(CH_3COO)_2·H_2O$ as a Cu source was dissolved in 5 mL DCDA/EG solution under ultrasonic for 15 min to form a blue Cu-DCDA/EG mixture. Subsequently, the blue Cu-DCDA/EG mixture was kept at temperature of 60 °C for 3 h in oven and a reddish-brown Cu-DCDA/EG complex was obtained. Next, 5 mL of Cu-DCDA/EG complex and DCDA/EG solution were added into a 30 mL of crucible and placed into a microwave reactor (700 W, M1-L213B, 2.45 GHz, Midea, China)[4], and then irradiated for 120 s. The as-resulted sample was denoted as $CuCN_{int.}$ and $CN_{int.}$ Finally, the obtained $CuCN_{int.}$ and $CN_{int.}$ were heated at a rate of 5 °C min^{-1} to reach 550 °C and maintained for 2 h in air, the resulting samples were the final product of $Cu_{SA}C_6N_6$ and CN_{mw}.

Synthesis of nanocomposite Cu-N-C/CN_{mw}. Firstly, 10 mg of as-prepared CN_{mw} was placed in a centrifuge tube and added into 1 mL of pure water to prepare the CN_{mw} nanosheets under ultrasonic for 1 h. Then, 5 mL of CN_{mw} nanosheets aqueous solution (1 mg/mL) and 30 mg of Cu-N-C SAzymes (0.15 mmol) were under ultrasonic for 30 min.

Characterization of $Cu_{SA}C_6N_6$. The X-ray power diffraction (XRD) patterns were measured by using an Ultima IV (Rigaku, Japan) with high intensity Cu-Kα radiation (λ=1.54178 Å). The Fourier transform infrared spectroscopy (FT-IR) was recorded with

a Nicolet iS10 FT-IR spectrometer (Thermo Fisher, USA) with the KBr tableting. The ESCALAB 250Xi electron spectrometer (Thermo Fisher, USA) was employed as the X-ray photoelectron spectrum (XPS) characterization with the peak of C1*s* (284.6 eV) as the reference for calibration. The NexION 1000G ICP Mass Spectrometer (PerkinElmer, USA) was employed as inductively coupled plasma mass spectrometry (ICP-MS) investigation. The ^{13}C and ^{1}H solid-state NMR spectrum were conducted on AVANCE III 400 MHz WB solid-state NMR spectrometer (Bruker, Germany). The scanning electron microscopy (SEM) images were taken on a FEI Inspect F50 (thermo Fisher, USA). The transmission electron microscopy (TEM) images were obtained from a JEM-2100F transmission electron microscopy (JEM, Japan) with accelerating voltage at 200 kV and the high-angle annular dark field scanning transmission electron microscopy (HAADF-STEM) images were performed by JEM-ARM300F GRAND ARM equipped with energy-dispersive X-ray spectroscopy (EDS) mapping at an accelerating voltage of 300 kV. The combustion elemental analysis was measured by an elementar vario el III (Elementar, German). The matrix-free laser desorption ionization time-of-flight mass spectrometry (LDI-TOF MS) was carried out by using a Smart Beam II Nd:YAG/355 nm laser operating at 2,000 Hz (ultrafleXtreme, Bruker, Germany). The UV-vis-NIR absorption spectrum was obtained from a Lambda750 spectrophotometer (PerkinElmer, USA). The electron spin resonance (ESR) spectra were obtained from EMXPlus spectrometer (Bruker, Germany) at room temperature. The Electrochemical impedance (EIS) were measured on Gamry Reference 600 potentiostat/galvanostat/ZRA (USA). The photoelectrochemical tests were carried on a CHI-600E electrochemical workstation (Shanghai, China) with a 150 W NBeT Xe lamp (Beijing, China). The X-ray absorption fine structures (XAFS) measurements were carried out on the sample at 21A X-ray nanodiffraction beamline of Taiwan Photon Source (TPS), National Synchrotron Radiation Research Center (NSRRC) and were provided for technical support by Ceshigo Research Service (www.ceshigo.com).

Peroxidase-like activity mimic of $Cu_{SA}C_6N_6$ under basic and light irradiation. The catalytic activity of $Cu_{SA}C_6N_6$ was evaluated by catalytic oxidation of ABTS in the

presence of H_2O_2. Typically, without the light radiation, 30 μL of $Cu_{SA}C_6N_6$ (10 mg mL^{-1}) was added firstly into 980 μL of 0.2 M HAc-NaAc buffer solution (pH 5.0), containing 10 μL of ABTS (50 mM in DMSO) and 10 μL of H_2O_2 (0.5 M). Then the test was started quickly at room temperature. During the 3 min incubation at 25 °C, the peroxidase-like activity was measured by monitoring the absorbance at 417 nm for the $ABTS_{ox}$ after filtration. While the light was on, the incubation was performed under a household white LED (λ=400-900 nm, 50 mW/cm^2).

Glucose detection under the different light intensity. Glucose was detected according to the previously reported literatures.[5] Briefly, 20 μL of 10 mg/mL GO_x and was added into 200 μL of glucose solution of different concentrations dissolved with 10 mM phosphate buffered saline (PBS, pH 7.0) and incubated at 37 °C for 30 min; Then, 720 μL of 0.2 M acetate buffer (pH = 5.0), 10 μL of 50 mM ABTS, and 30 μL of the $Cu_{SA}C_6N_6$ stock solution, were added into the above 220 μL glucose reaction solution. Next, the mixed solution was incubated in a 25 °C water bath. During the 3 min incubation at 25 °C under basic condition, the final reaction solution was used to perform the absorption spectroscopy measurement at 417 nm for the $ABTS_{ox}$ after filtration. Similarly, the incubation was performed under a household white LED (λ=400-900 nm, 30 mW/cm^2 and 50 mW/cm^2). The various light intensity was achieved by modulating the distance between light source and sample. The GOx image in Figure 6a was created using the structure-related data from RCSB.org (PDB ID: 1CF3) by Visual Molecular Dynamics (VMD, revision 1.9.3).

Free radical identification. The reactive oxygen species (ROS) generated in the oxidation of ABTS upon the basic and gain reaction were identified by comparing the reactivity in the absence or presence of ROS scavengers (SOD and isopropanol scavenge superoxide and hydroxyl radical, respectively). Upon the light off, 30 μL of $Cu_{SA}C_6N_6$ (10 mg mL^{-1}) was added firstly into 950 μL of 0.2 M HAc-NaAc buffer solution (pH 5.0), containing 10 μL of SOD (2 mg mL^{-1}; or 10 μL of 1 M isopropanol), 10 μL of ABTS (50 mM in DMSO) and 10 μL of H_2O_2 (0.5 M). Then the test was started quickly at room temperature. While the light was on, the incubation was performed

under a household white LED (λ=400-900 nm, 50 mW/cm^2).

Monitoring ROS species by ESR. The reactive oxygen species (ROS) generated with the basic and gain reaction were also identified by ESR measurements with ROS trapping agent (TEMP ($^1O_2$) and DMPO (·OH, $O_2^{\cdot-}$). Upon the light off, 30 μL of $Cu_{SA}C_6N_6$ (10 mg mL^{-1}) was added firstly into 960 μL of 0.2 M HAc-NaAc buffer solution (pH 5.0), containing 10 μL of TEMP (50 mM; or 10 μL of 50 mM DMPO), 10 μL of H_2O_2 (0.5 M). Then the aqueous solution (1 mL) was incubated for 5 min. Then, the mixture after filtration was transferred into a capillary tube for ESR measurements. When the light was on, the incubation was performed under a household white LED (λ=400-900 nm, 50 mW/cm^2).

Synchronous illumination X-ray photoelectron spectroscopy (SI-XPS). The SI-XPS tests were performed on a XPS instrument (ESCALAB 250Xi) equipped with a 300 W Xe arc lamp as illumination source for providing the simulated solar-light with full spectrum. During the measurements process, the changes of XPS spectra were recorded by controlling light on or off at given time intervals. The samples were dispersed in water, deposited on substrates, and dried for 12 h. After that, the obtained samples were put in pretreatment chamber of XPS equipment for 12 h to remove the physically absorbed water molecules. Finally, the samples were transferred to the analysis chamber to perform the SI-XPS measurements.

Computational methods. Density functional theory calculations were performed using Gaussian 16 (revision C.02).[6] The ground-state geometries were optimized at a typical M06-2X/6-31G(d) level. All the optimized structures were real minima on the potential energy surface by means of frequency calculations. For the structure model anchored with/without a Cu atom, the charge of the system was set to +1/ 0 according to the experimental results, and the spin multiplicity was set to 1. Based on the optimized structures, electron excitations were calculated by means of the time-dependent density functional theory method. For this purpose, the first 50 excited states were calculated using M06-2X (54% Hartree-Fock function)[7] with def2-TZVP basis set in the gas

phase.[8] To evaluate the reliability of the M06-2X hybrid functional in our system, the ground-state geometries (the 6-31G(d) basis set) and electron excitation (the def2-TZVP basis set) using PBE0 (25% Hartree-Fock function)[9, 10] and ωB97XD (22.2% Hartree-Fock function)[11, 12] hybrid functionals were also calculated (Supplementary Fig. 40), which gave the similar results.

The absorption spectra, the excited state of the electron-hole distribution[13] and the charge transfer spectra (CTS) were analyzed by Multiwfn (revision 2.8(dev)),[14] and the images of structures and isosurfaces of the electron-hole distribution were obtained from Visual Molecular Dynamics (VMD, revision 1.9.3). Detailed formulas for calculating the CTS were described in the study by Lu et al.[15] The CTS were calculated using Mulliken population analysis of the inter-fragment charge transfer term for each excited state. The absorption spectra and CTS were obtained using a Gaussian broadening function with a full-width-at-half-maximum of 0.67 eV.

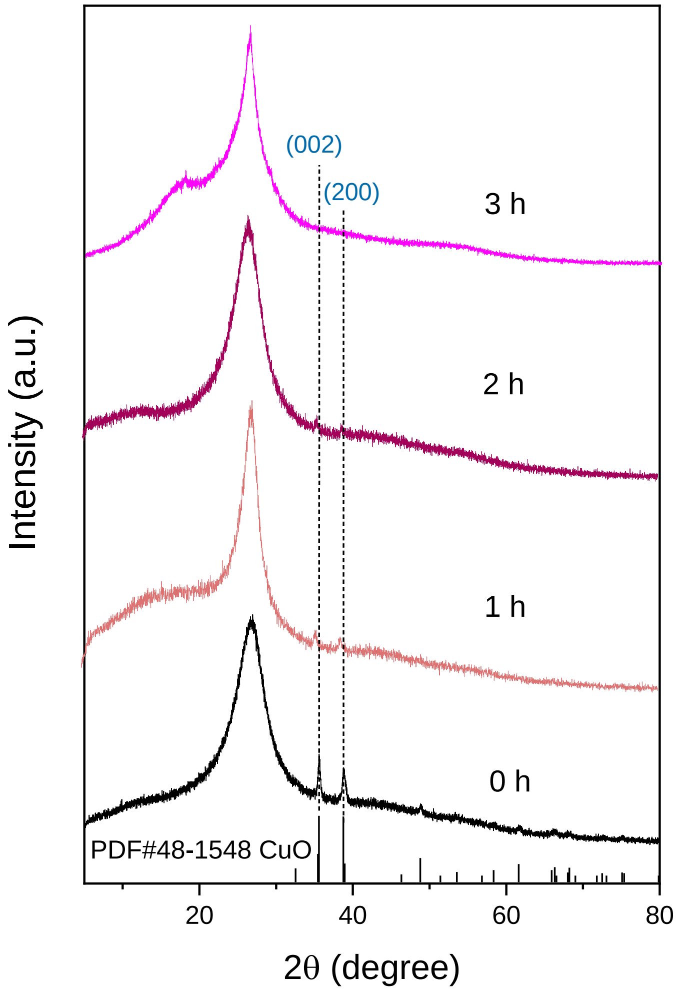

Supplementary Fig. 1 XRD patterns of Cu-$CN_{int.}$ prepared from Cu/DCDA with different pre-coordination time (0, 1, 2 and 3 h).

As seen in **Supplementary Fig. 1**, XRD patterns of Cu-$CN_{int.}$ prepared from Cu/DCDA-0 h not only had a peak at 26.7°, but also showed series of peaks at 35.7°, 38.8°and 48.9°, according to the PDF card of CuO.[16] In contrast, with the extension of the coordination time for 3 h, the CuO phase by microwave-assisted condensation of pre-coordinated DCDA-Cu complex was negligible. The result indicated the pre-coordinated Cu-DCDA complex in EG prevented the formation of metal or metallic oxide in Cu-$CN_{int.}$.

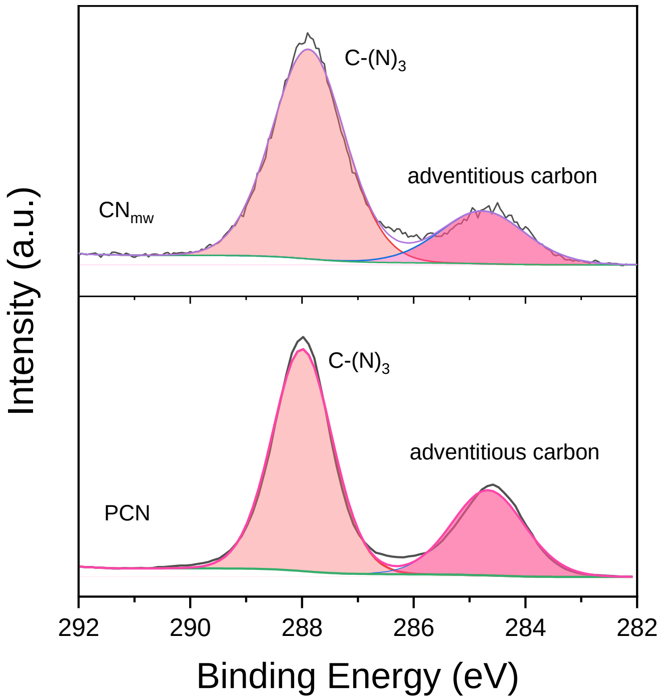

Supplementary Fig. 2 High-resolution C 1*s* XPS spectra of PCN and CN_{mw}.

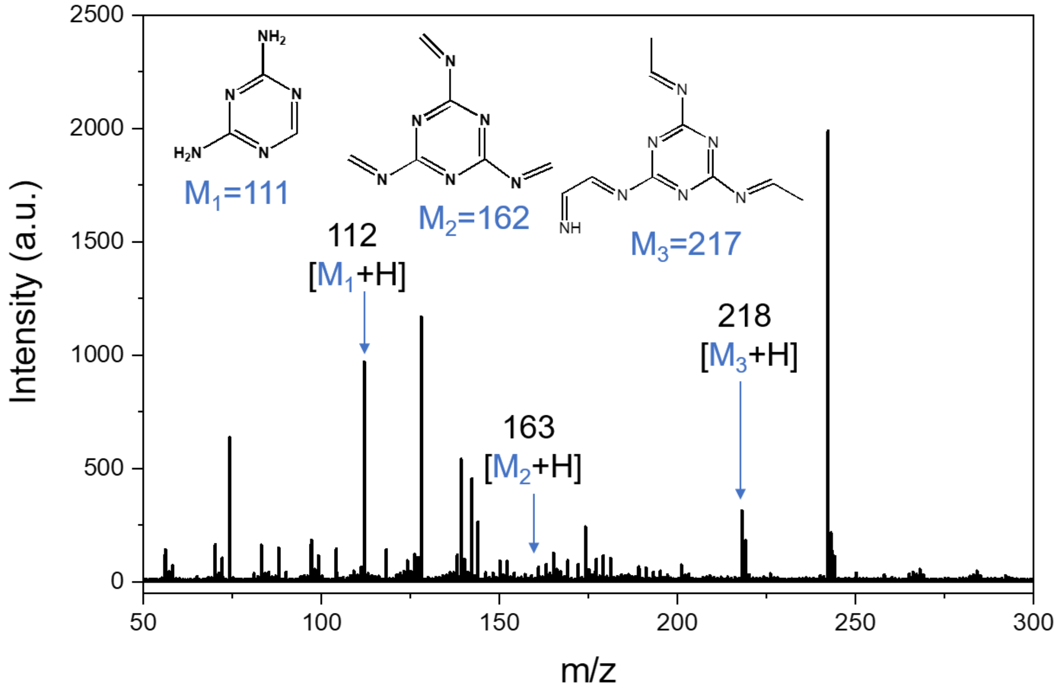

Supplementary Fig. 3 Matrix-free LDI-TOF mass spectrum of $Cu_{SA}C_6N_6$.

Supplementary Fig. 3 illustrated m/z peaks including [M + H$^+$] of 112 assigning to $C_3N_5H_5$ (M_1, calc.: 111), m/z [M + H$^+$] of 163 attributable to $C_6N_6H_6$ (M_2, calc.: 162), and m/z [M + H$^+$] of 218 attributable to $C_9N_7H_{11}$ (M_3, calc.: 217)

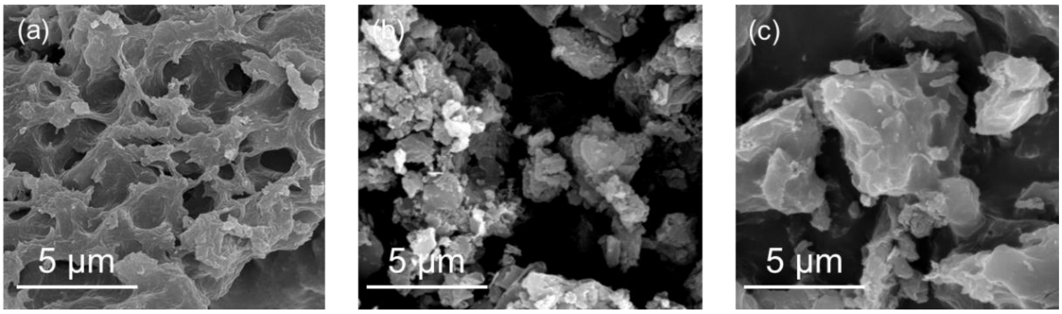

Supplementary Fig. 4 SEM images of (a) $Cu_{SA}C_6N_6$, (b) PCN and (c) CN_{mw}.

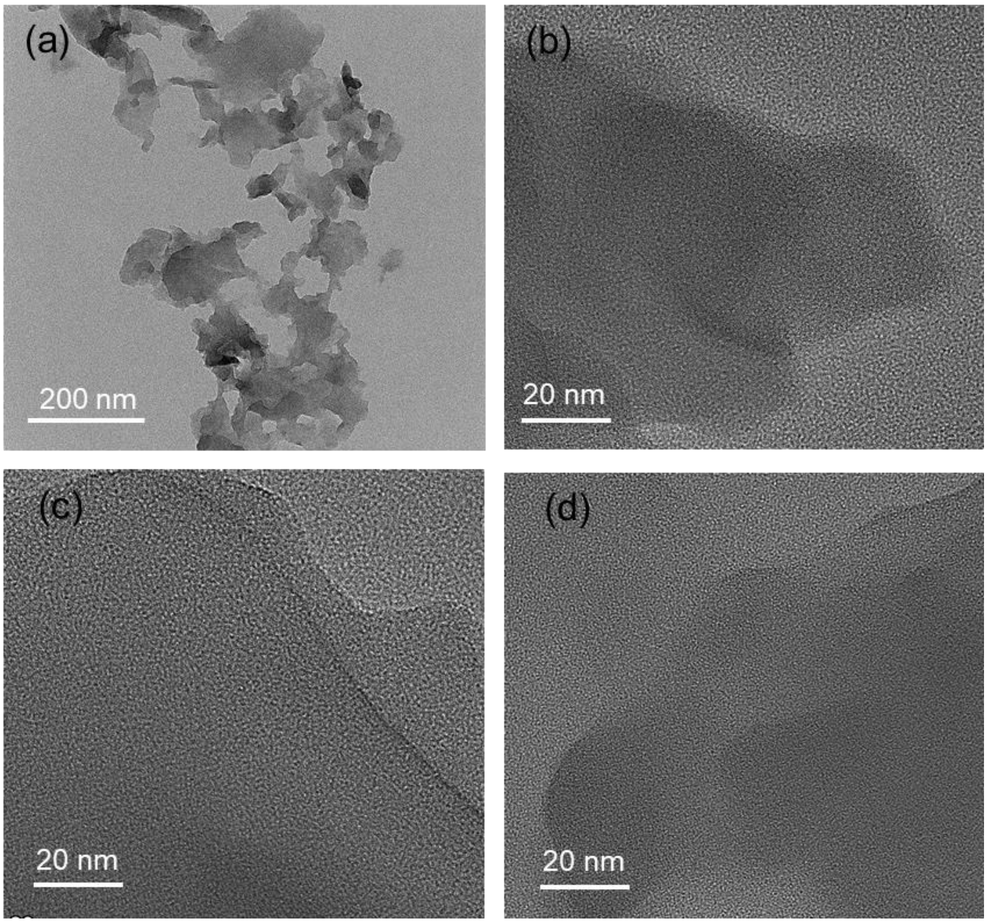

Supplementary Fig. 5 High-resolution TEM images of $Cu_{SA}C_6N_6$ with different magnifications at scale bars of (a) 200 nm and (b, c, d) 20 nm.

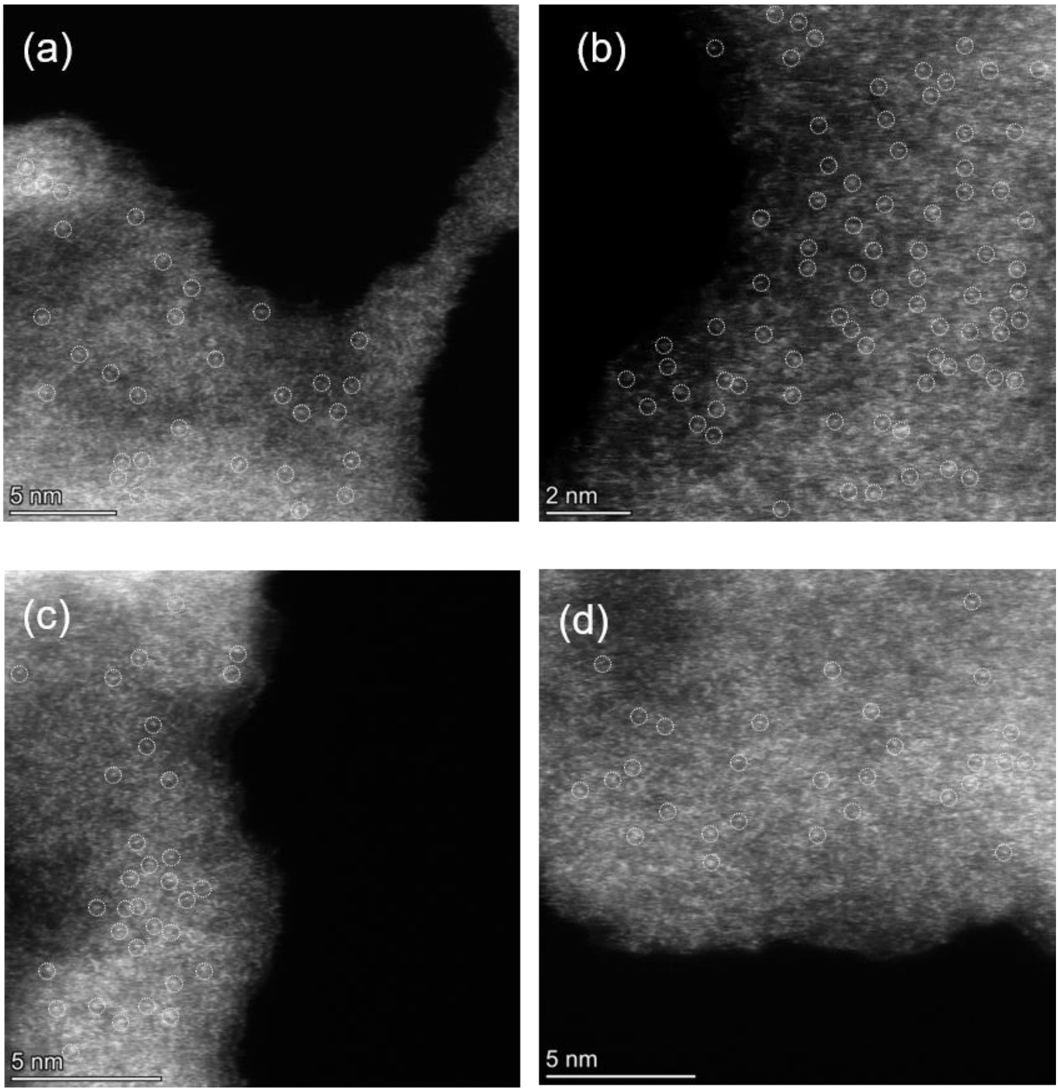

Supplementary Fig. 6 HAADF-STEM images (a, b, c, d) of $Cu_{SA}C_6N_6$ recorded at different areas.

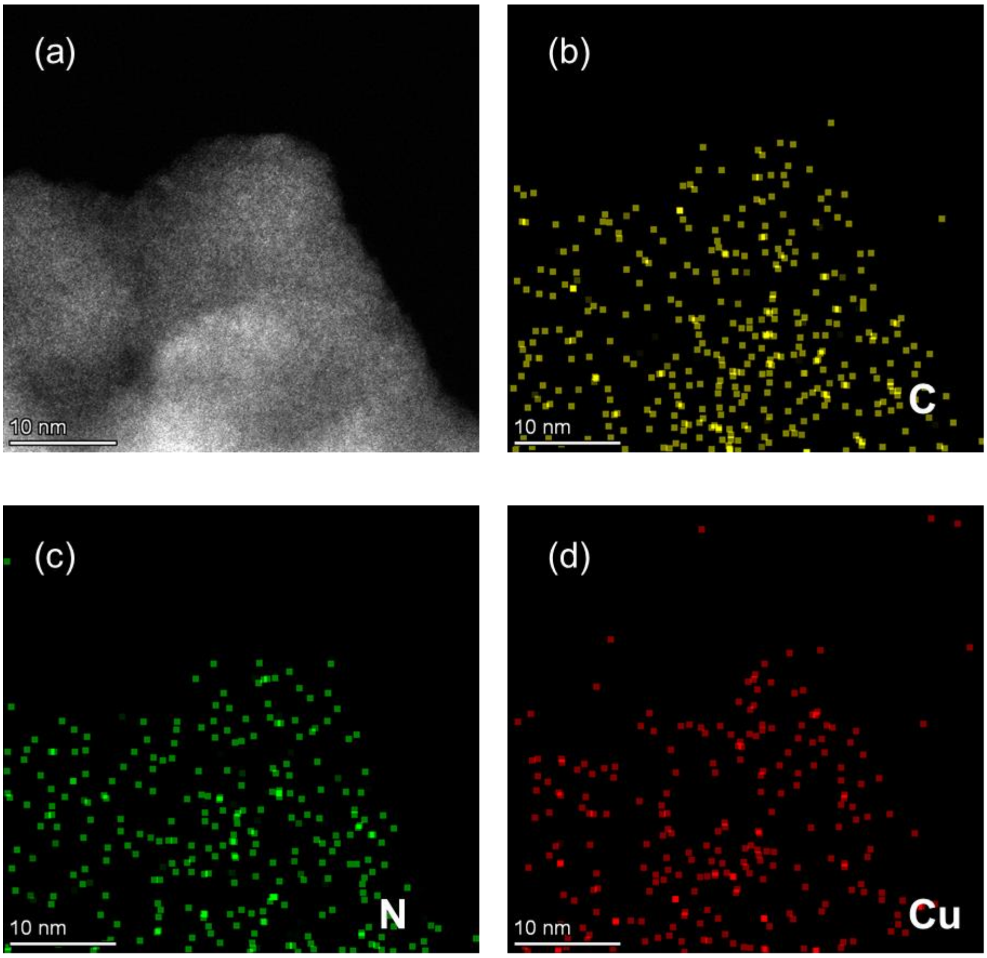

Supplementary Fig. 7 TEM image (a) and corresponding high-resolution STEM-EDS elemental mapping images of (b) C, (c) N and (d) Cu species of $Cu_{SA}C_6N_6$.

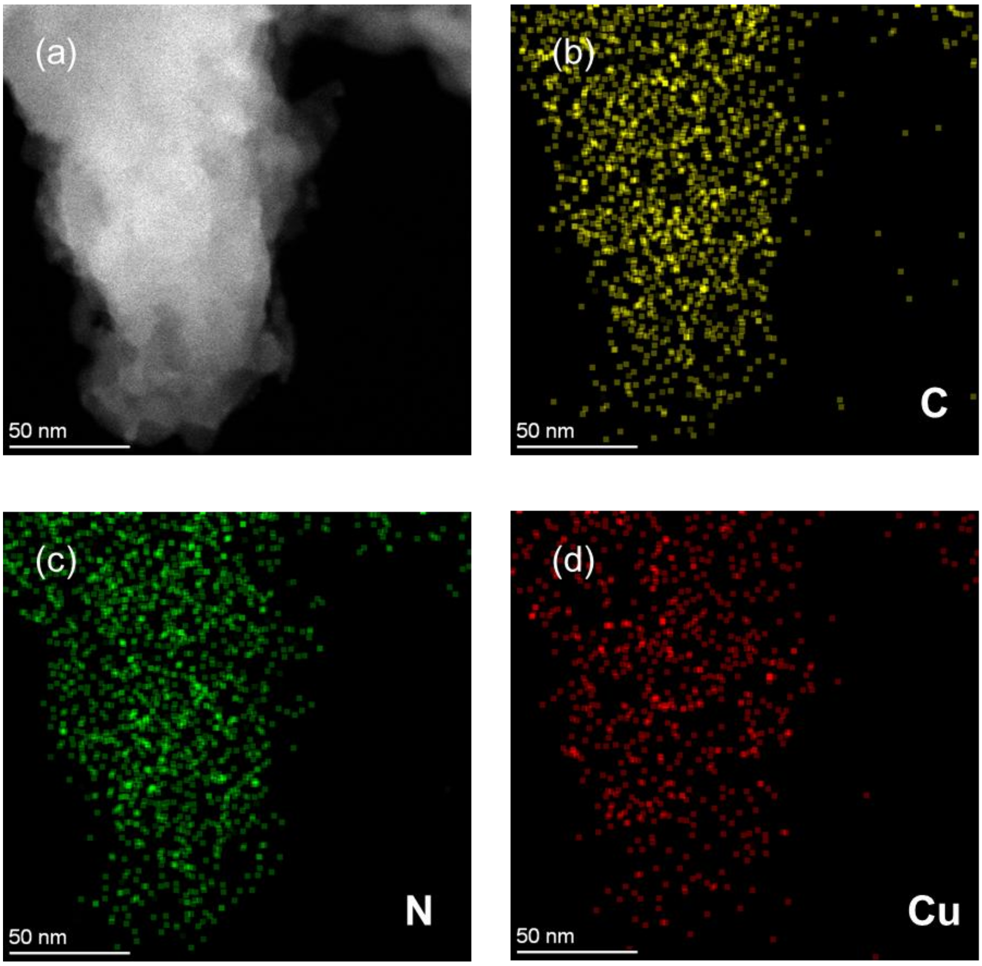

Supplementary Fig. 8 TEM image (a) and corresponding STEM-EDS elemental mapping images of (b) C, (c) N and (d) Cu species of $Cu_{SA}C_6N_6$ in other area.

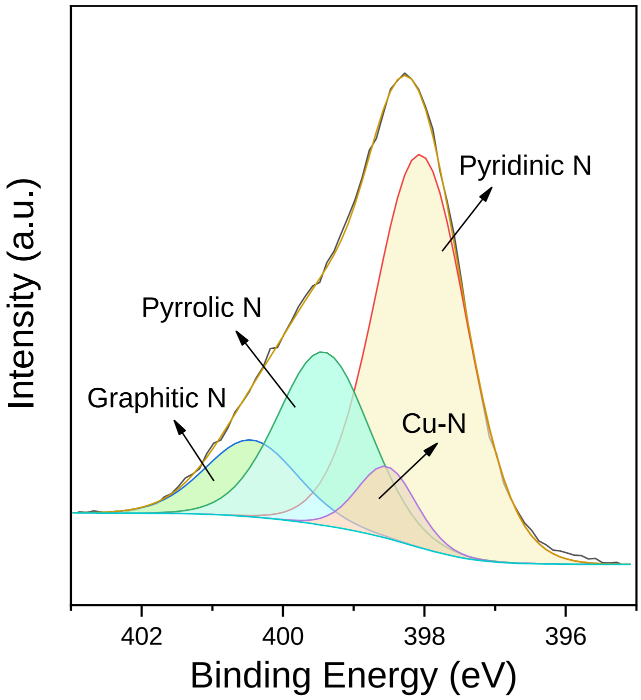

Supplementary Fig. 9 High-resolution N 1*s* XPS spectrum of $Cu_{SA}C_6N_6$.

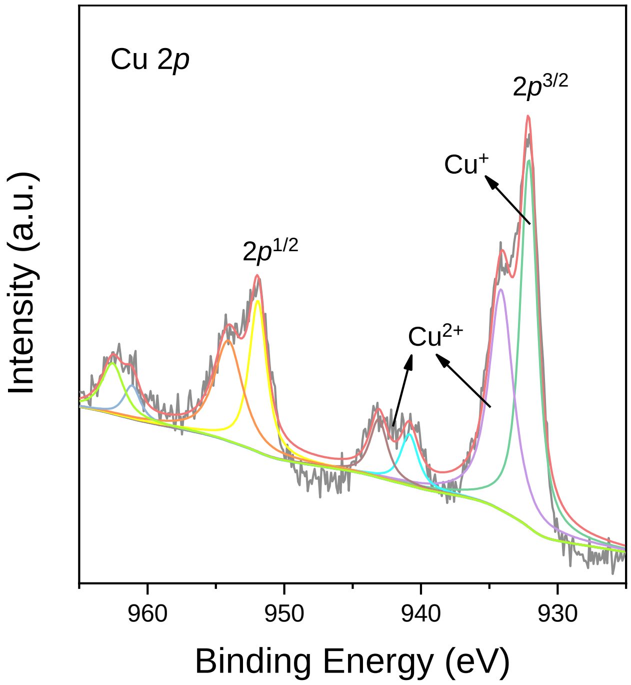

Supplementary Fig. 10 High-resolution Cu 2*p* XPS spectrum of $Cu_{SA}C_6N_6$.

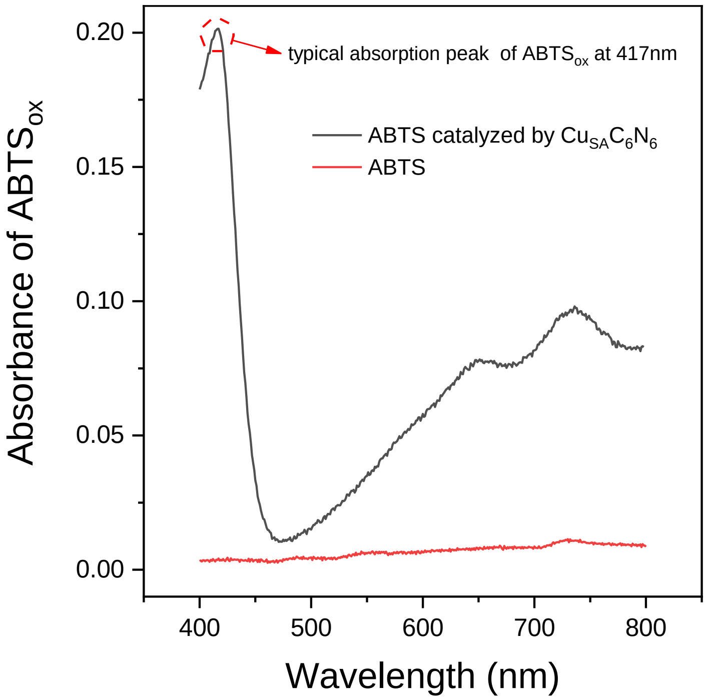

Supplementary Fig. 11 UV-vis absorption spectra of ABTS and that catalyzed by $Cu_{SA}C_6N_6$ in the presence of H_2O_2.

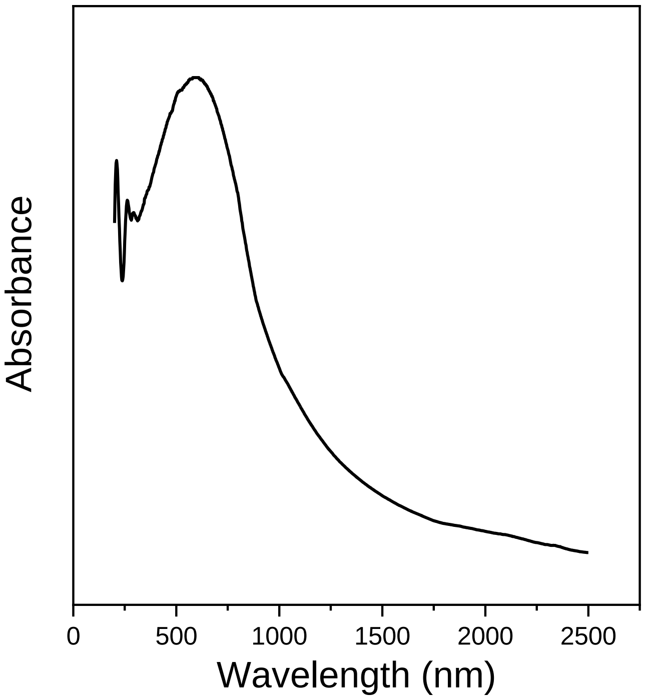

Supplementary Fig. 12 Ultraviolet-visible-near-infrared absorption spectrum of $Cu_{SA}C_6N_6$.

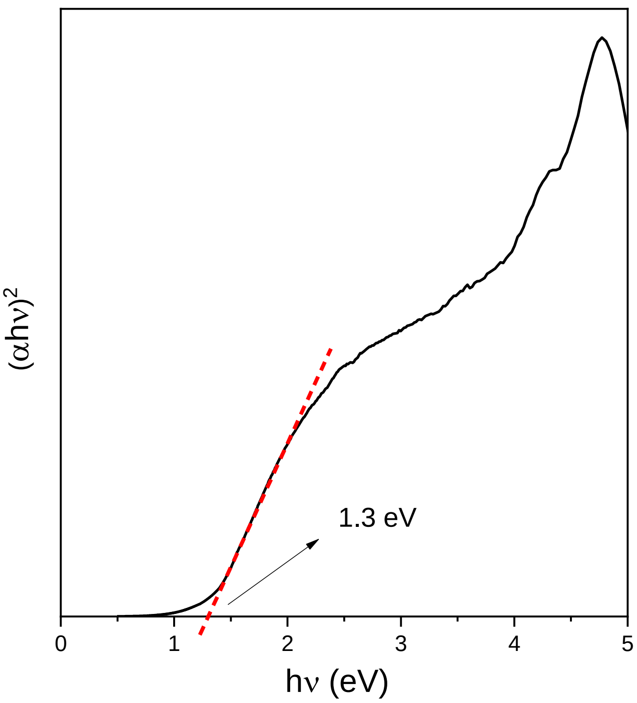

Supplementary Fig. 13 Kubelka-Munk plot of $Cu_{SA}C_6N_6$ powders.

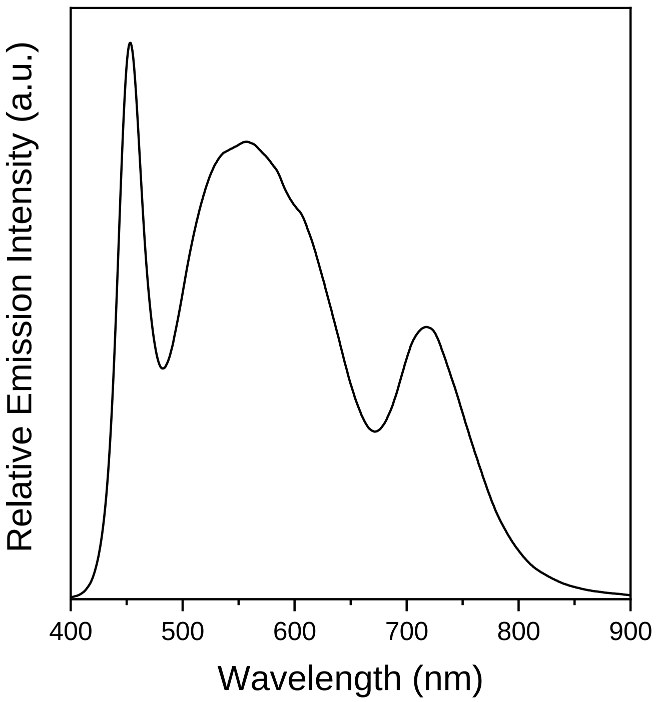

Supplementary Fig. 14 Emission spectrum of the white household LED lamp in this study.

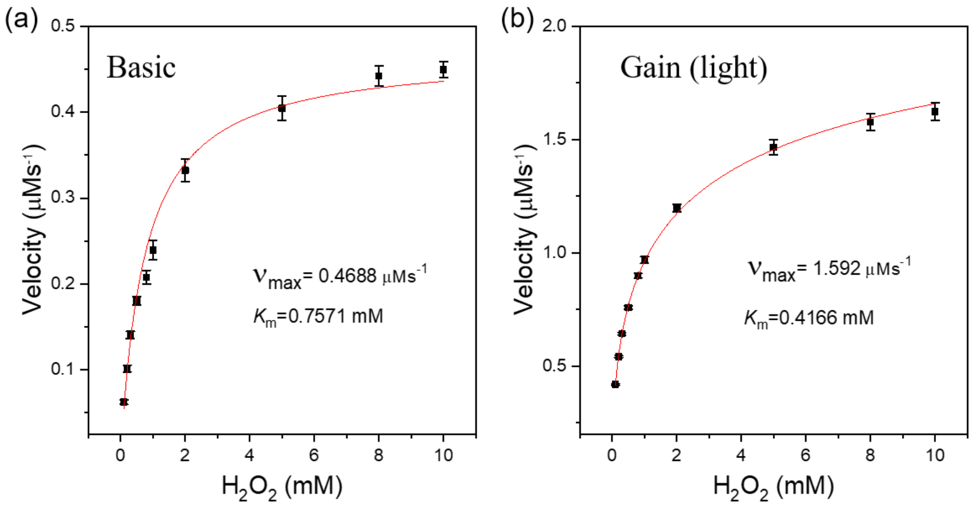

Supplementary Fig. 15 Peroxidase-like velocity of $Cu_{SA}C_6N_6$ in (a) basic and (b) gain (light) reactions at 25 ^oC. Error bars represent the standard error derived from three independent measurements.

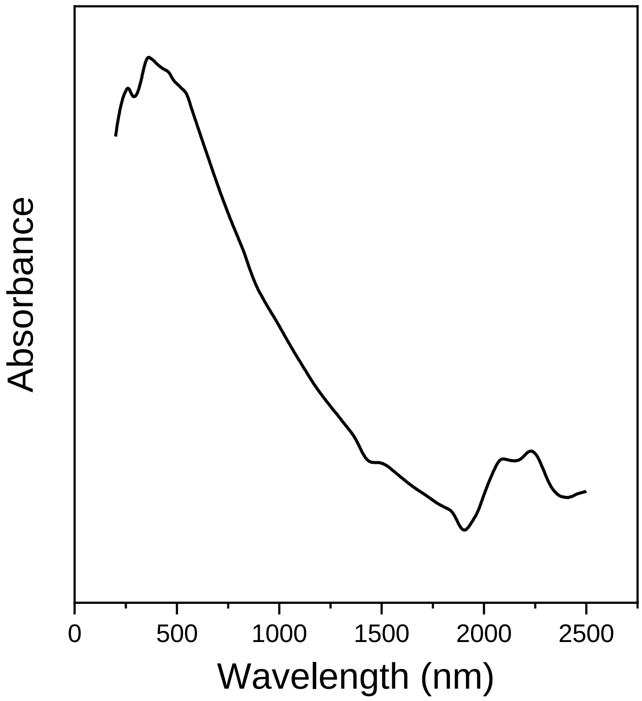

Supplementary Fig. 16 Ultraviolet-visible-near-infrared absorption spectrum of CN_{mw}.

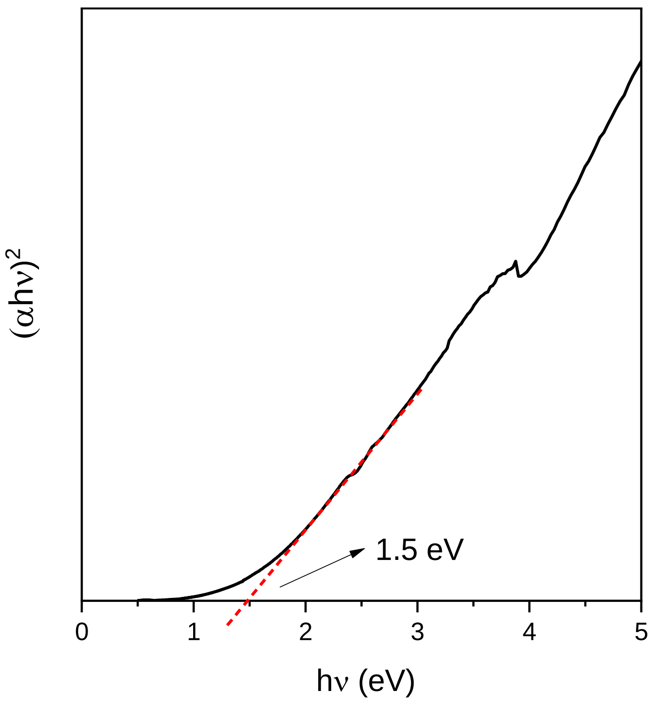

Supplementary Fig. 17 Kubelka-Munk plot of CN_{mw} powders.

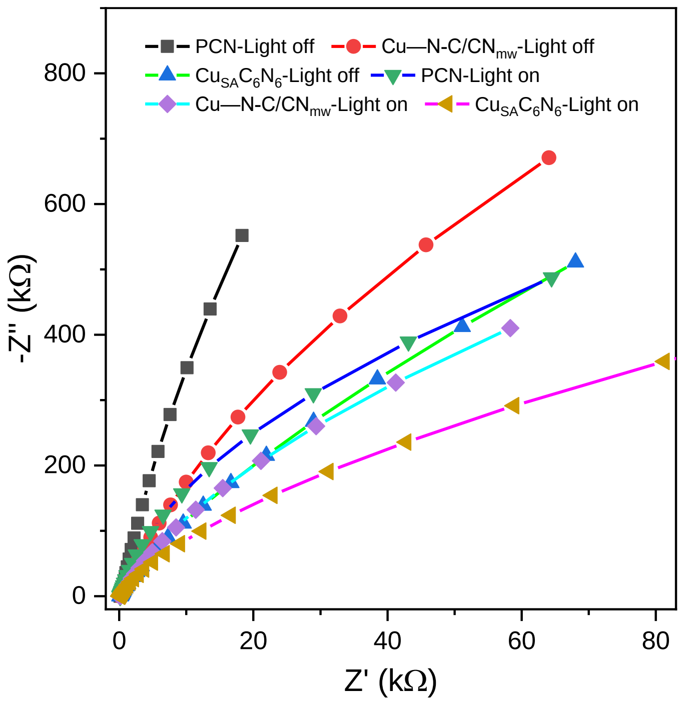

Supplementary Fig. 18 Nyquist plots of $Cu_{SA}C_6N_6$, Cu-N-C/CN_{mw} and PCN with/without light illumination.

As shown in **Supplementary Fig. 18**, a much smaller impedance of $Cu_{SA}C_6N_6$ was observed, compared to that of Cu-N-C/CN_{mw} and PCN under the basic condition. When the light was on, the impedance of the three catalysts significantly decreased, indicating a remarkable increase of the electron conductivity. Interestingly, $Cu_{SA}C_6N_6$ still exhibited the minimum impedance, which demonstrated the fastest charge transfer kinetics based on a specialized charge transfer pathway.

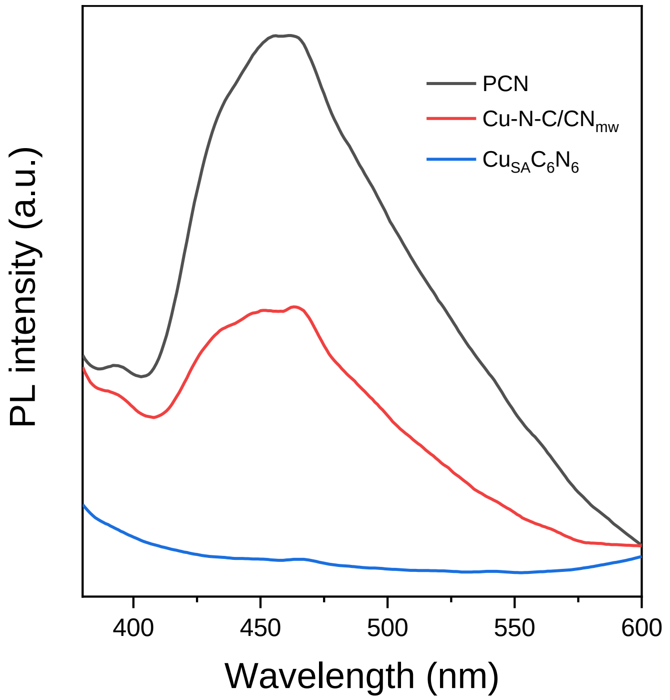

Supplementary Fig. 19 Photoluminescence (PL) spectra of $Cu_{SA}C_6N_6$, Cu-N-C/CN$_{mw}$ and PCN.

As shown in **Supplementary Fig. 19**, the PL intensity of $Cu_{SA}C_6N_6$ was virtually invisible in comparison with Cu-N-C/CN$_{mw}$ and PCN, indicating an increased amount of non-radiative transition presumably by multiple pathways of charge transfer.

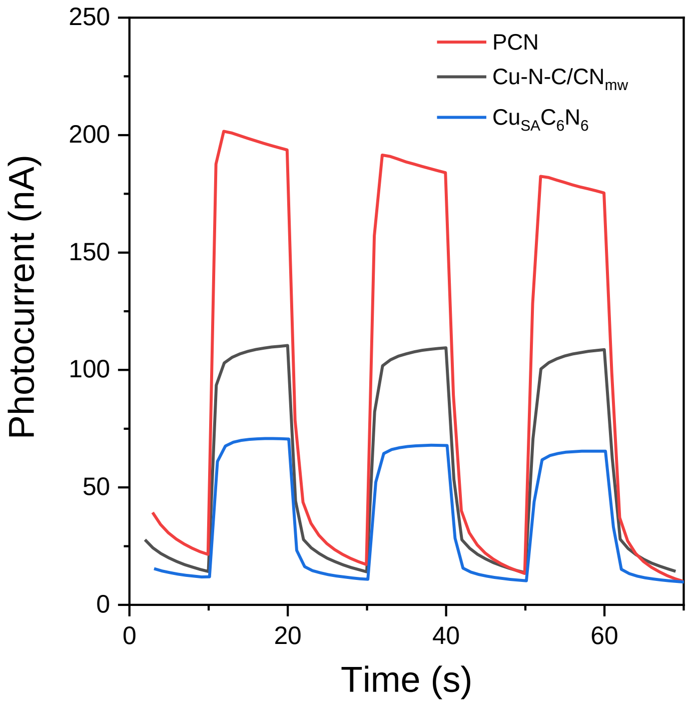

Supplementary Fig. 20 Photocurrent of $Cu_{SA}C_6N_6$, Cu-N-C/CN_{mw} and PCN under chopped light.

Photoelectrochemical (PEC) experiments were carried on a CHI-600E electrochemical workstation with a three-electrode system containing a Pt wire as counter electrode, an Ag/AgCl (saturated KCl) as reference electrode and $Cu_{SA}C_6N_6$, Cu-N-C/CN_{mw}, or PCN as working electrode. The electrolytes contained 0.1 M KCl and 30% Triethanolamine (TEOA), unless otherwise specified. The PEC experiments were also employed to evaluate the charge-transfer properties of $Cu_{SA}C_6N_6$, Cu-N-C/CN_{mw} and PCN. It was found the photocurrent of PCN based photoelectrode was the highest during light on/off cycles, while the photocurrents of Cu-N-C/CN_{mw} and $Cu_{SA}C_6N_6$ were lower (**Supplementary Fig. 20**), revealing that the Cu single atoms emerged as electron acceptors in C_6N_6 framework.

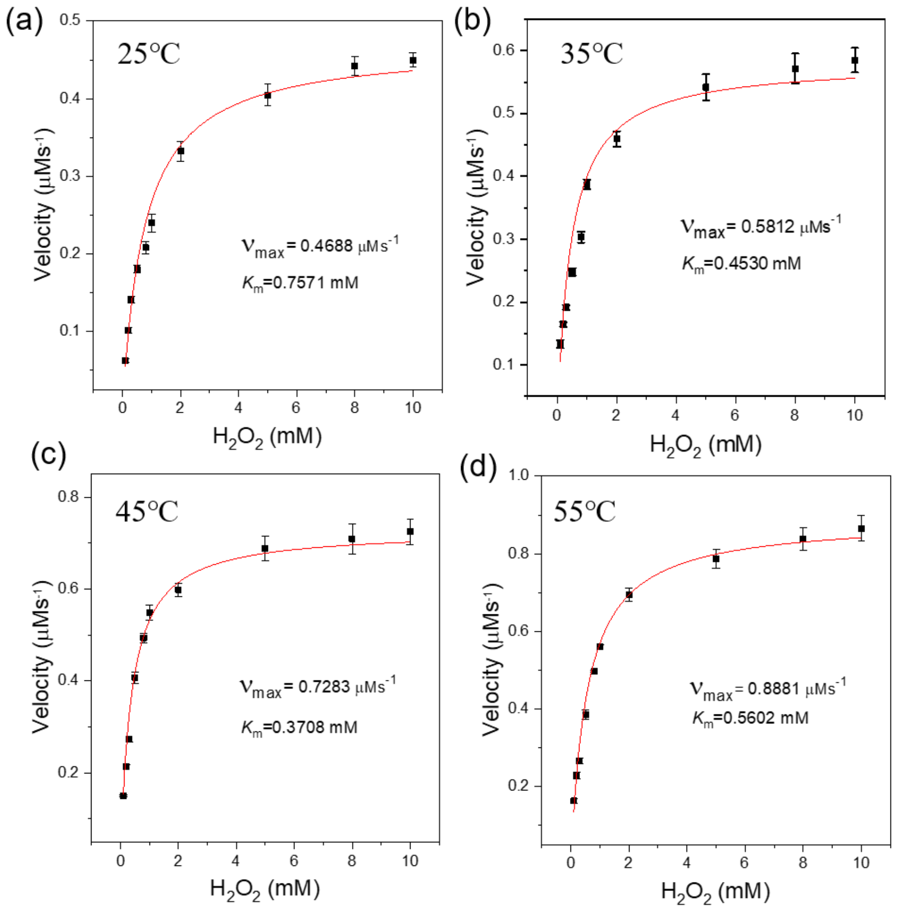

Supplementary Fig. 21 Peroxidase-like velocity of $Cu_{SA}C_6N_6$ in basic reaction at different temperatures. (a) 25 °C, (b) 35 °C, (c) 45 °C and (d) 55 °C. Error bars represent the standard error derived from three independent measurements.

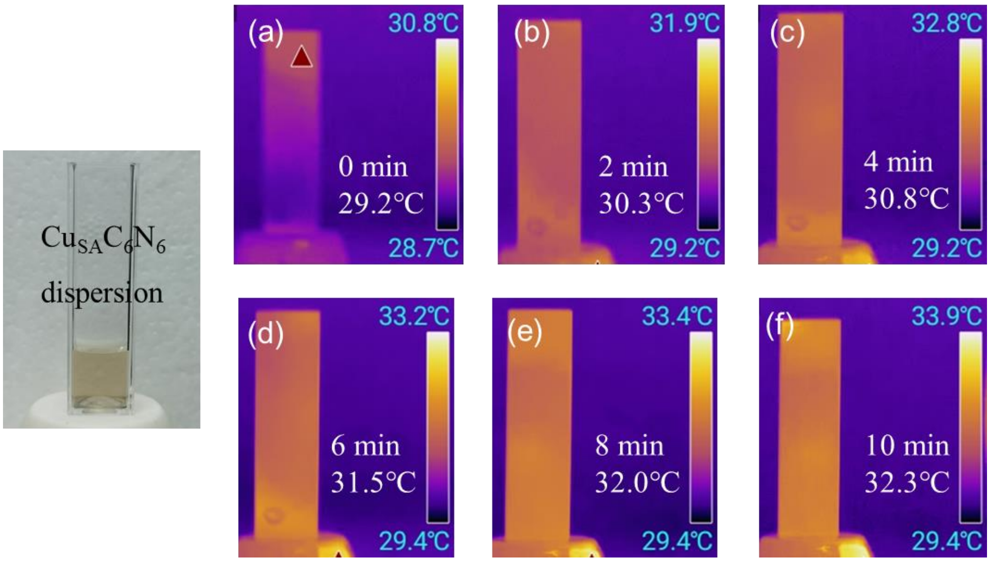

Supplementary Fig. 22 Optical and thermal images of $Cu_{SA}C_6N_6$ dispersion showing the photothermal effect with irradiation time for (a) 0 min, (b) 2 min, (c) 4 min, (d) 6 min, (e) 8 min, and (f) 10 min.

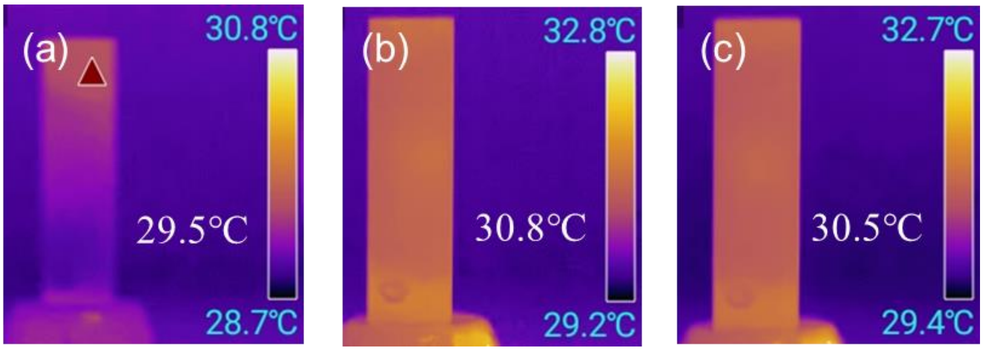

Supplementary Fig. 23 Thermal images of $Cu_{SA}C_6N_6$ dispersion at (a) room temperature, and that at an increase of temperature by (b) a heater and (c) light irradiation.

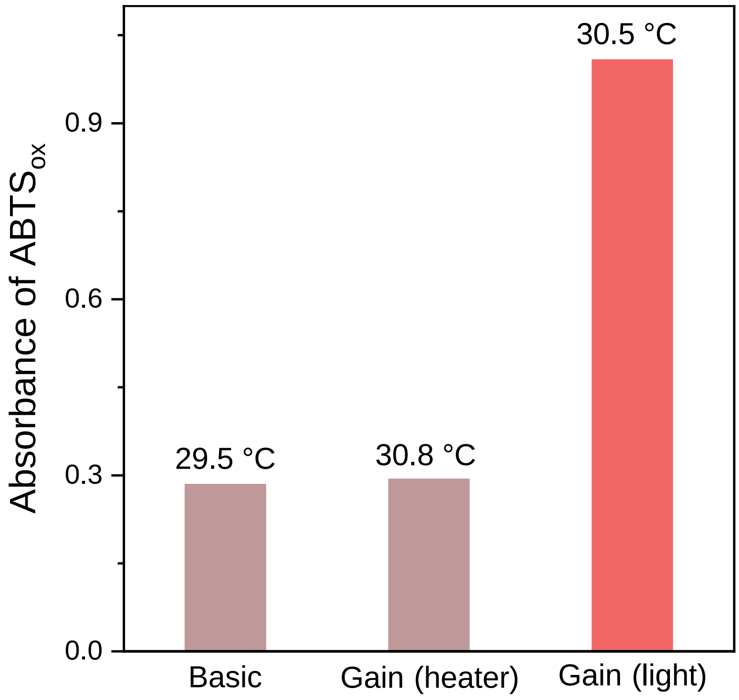

Supplementary Fig. 24 Absorbance of $ABTS_{ox}$ catalyzed by $Cu_{SA}C_6N_6$ under same temperature tuned by a heater and light irradiation.

As shown in **Supplementary Fig. 23** and **Supplementary Fig. 24**, the temperature of reactors was tuned using a heater to the same value as that by light irradiation. The enhancement of peroxidase-like activity was negligible under the same increase of temperature, confirming the photothermal-induced gain effect here was marginal.

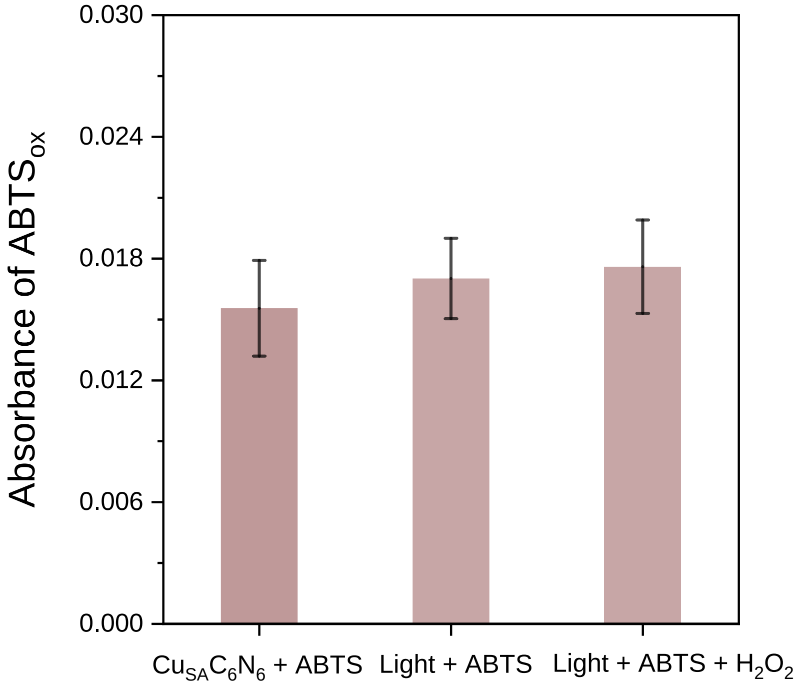

Supplementary Fig. 25 Absorbance of $ABTS_{ox}$ under control experiments. Error bars represent the standard error derived from three independent measurements.

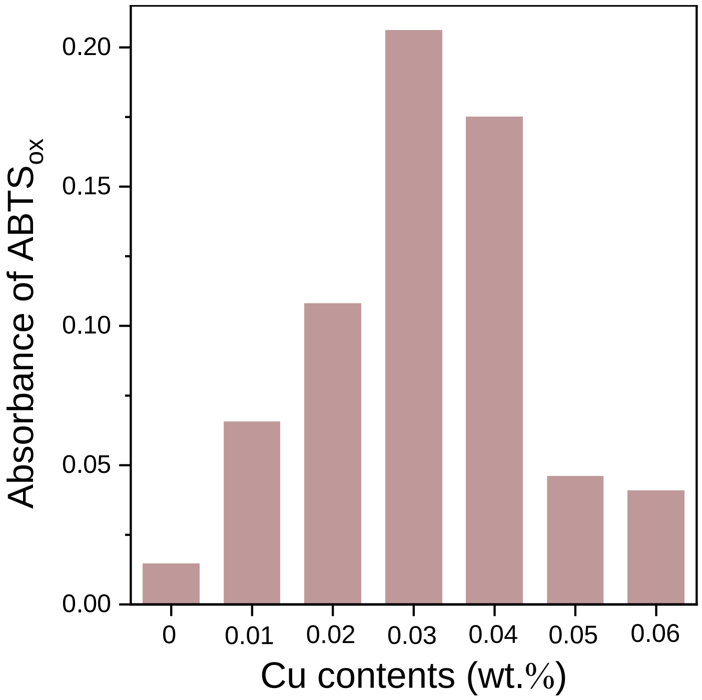

Supplementary Fig. 26 Absorbance of $ABTS_{ox}$ catalyzed by $Cu_{SA}C_6N_6$ with various Cu contents in the presence of H_2O_2.

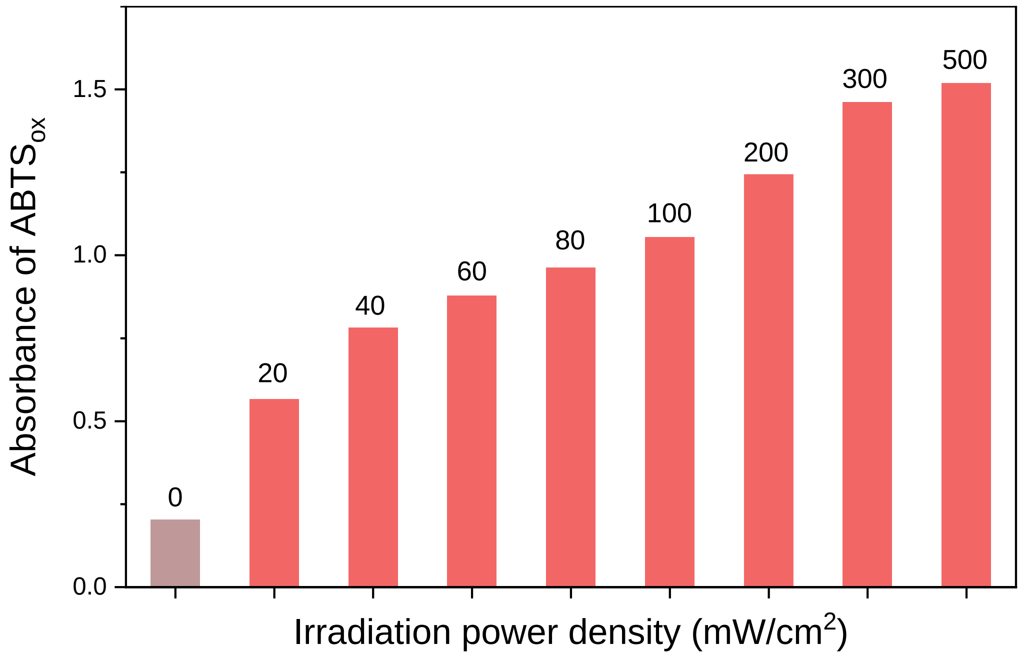

Supplementary Fig. 27 Absorbance of $ABTS_{ox}$ catalyzed by $Cu_{SA}C_6N_6$ under different irradiation intensity.

As shown in **Supplementary Fig. 27**, the photocatalytic performance of $Cu_{SA}C_6N_6$ could be facilely enhanced approximately 8 times by tuning the irradiation power density.

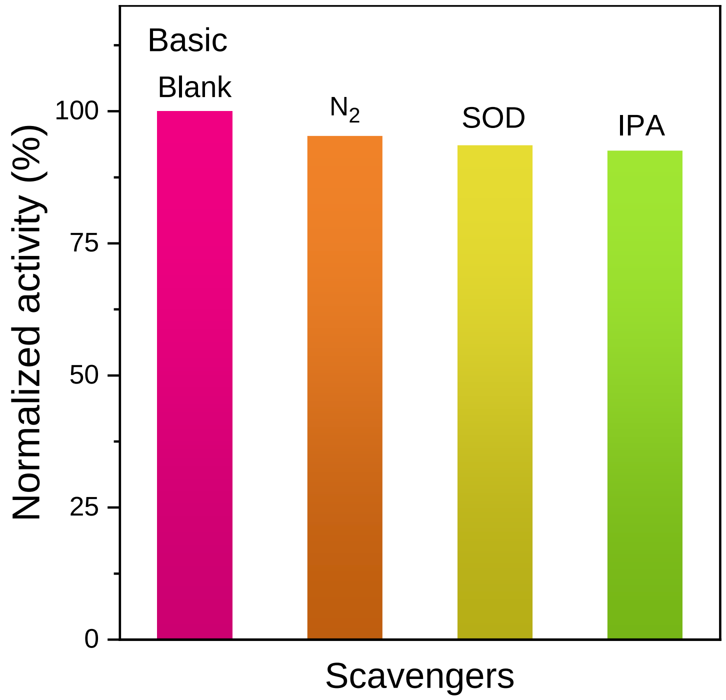

Supplementary Fig. 28 Peroxidase-like activity of $Cu_{SA}C_6N_6$ in the presence of different scavengers in basic reaction.

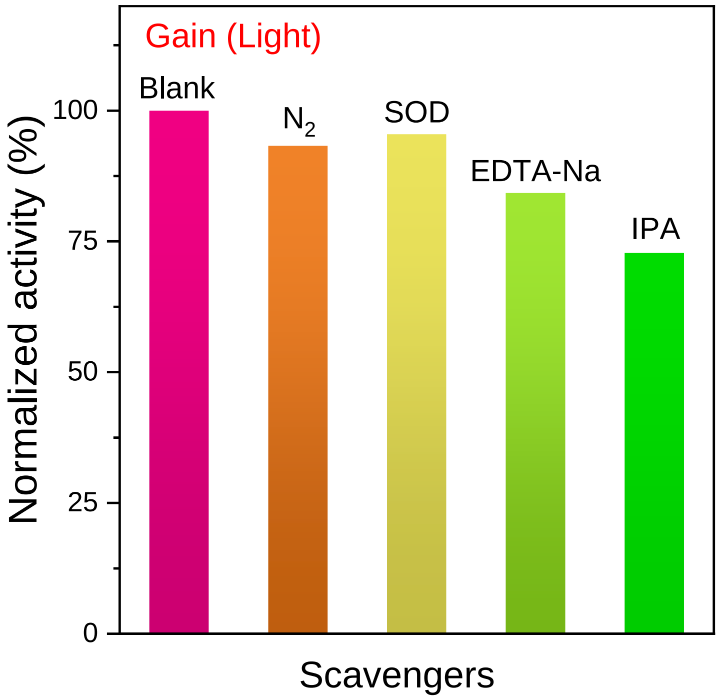

Supplementary Fig. 29 Peroxidase-like activity of $Cu_{SA}C_6N_6$ in the presence of different scavengers in the gain reaction.

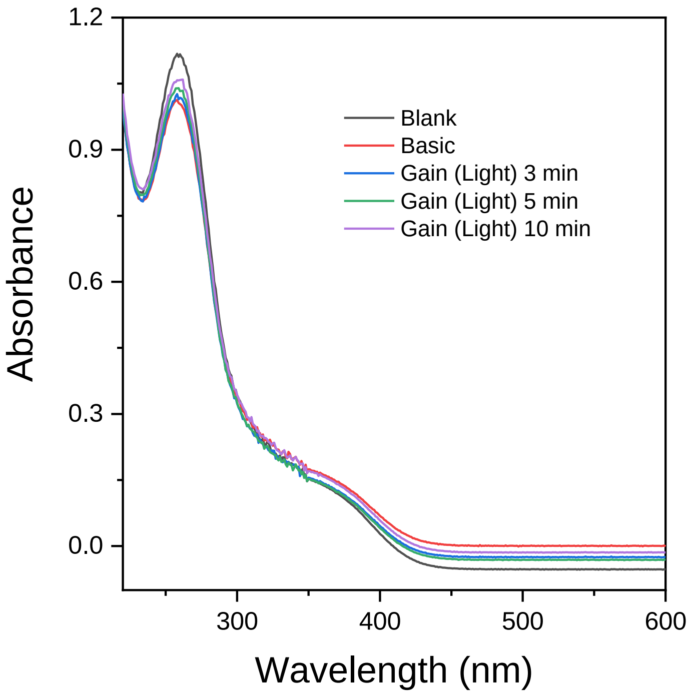

Supplementary Fig. 30 UV-vis absorption spectra of NBT_{re} catalyzed by $Cu_{SA}C_6N_6$ under basic and gain reaction.

NBT, a widely used probe for the detection of SOD-like activity, could be specifically reduced by $O_2^{\cdot-}$ to produce a wide absorption spectrum from 450 nm to 700 nm (typically centered at 550 nm).[17] As shown in **Supplementary Fig. 30**, the absorbance of NBT_{re} at 550 nm was negligible under the basic and gain reaction, confirming the generation of $O_2^{\cdot-}$ in catalytic oxidation of TMB process here was marginal.

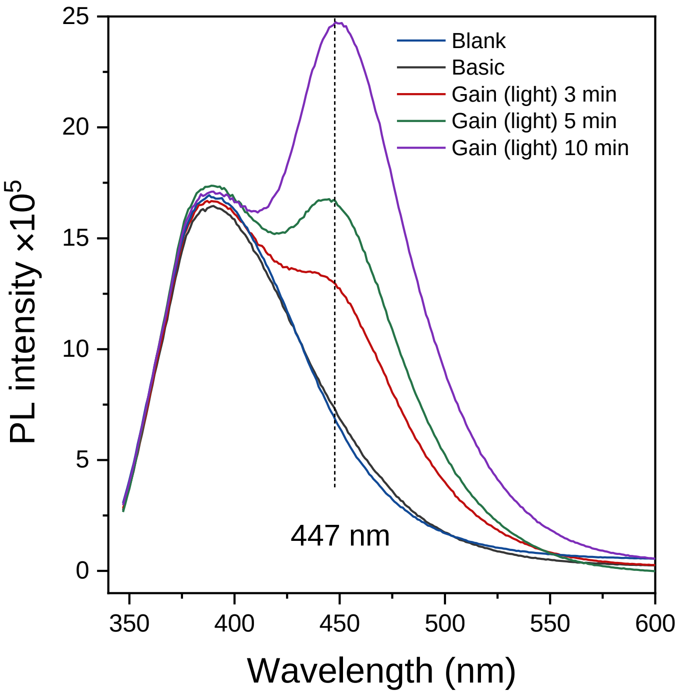

Supplementary Fig. 31 PL spectra of umbelliferone catalyzed by $Cu_{SA}C_6N_6$ in basic and gain reaction.

Since coumarin has no absorption of the excitation light, this molecule was employed to generate the fluorescent product for detecting ·OH radicals in solution.[18] As shown in **Supplementary Fig. 31**, the fluorescence emission peak of umbelliferone at 447 nm was remarkable, confirming the existence of ·OH in catalytic oxidation of ABTS under the gain reaction.

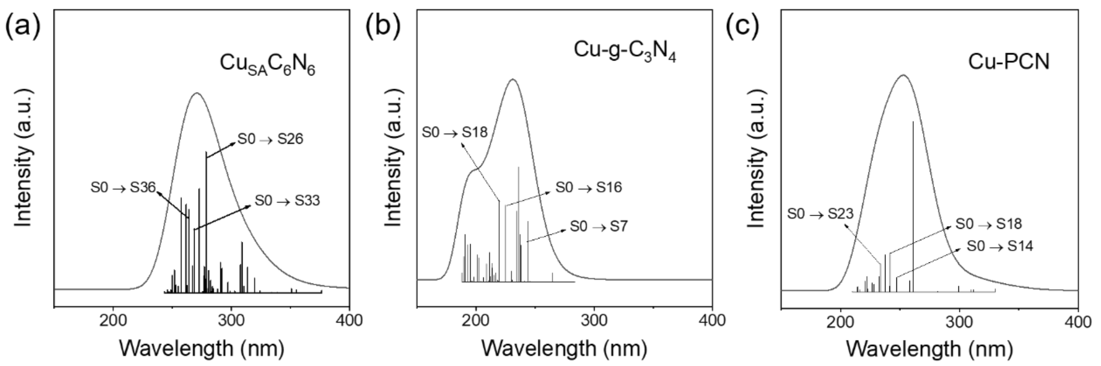

Supplementary Fig. 32 Simulated absorption spectra (curve) based on the first 50 excited states (vertical line) of (a) $Cu_{SA}C_6N_6$, (b) Cu-g-C_3N_4 and (c) Cu-PCN. The three most significant delocalization excitations of each system are marked. Electron excitations were calculated with M06-2X/def2-TZVP level based on optimized ground-state geometries.

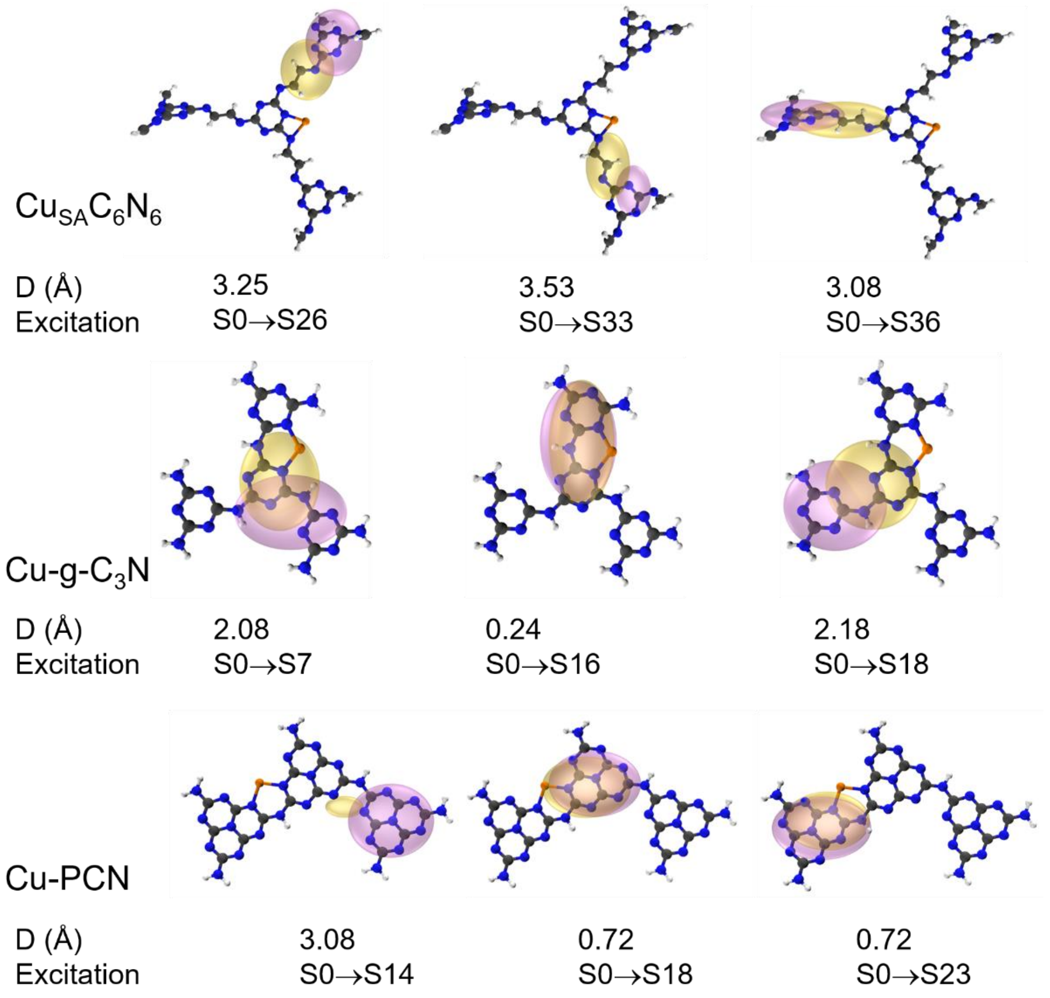

Supplementary Fig. 33 Isosurfaces of hole and electron distribution of the three highest intensity of delocalization excitations for $Cu_{SA}C_6N_6$, Cu-g-C_3N_4, and Cu-PCN. The corresponding excitation and electron-charge center-of-mass distance (D) were marked below the picture. Calculated smooth description of electron (yellow isosurface) and hole (pink isosurface) spatial population distributions (isovalue = 0.001 au), respectively. Carbon atoms (black), Nitrogen atoms (blue), Hydrogen atoms (white), Cu atoms (orange). Electron excitations were calculated with M06-2X/def2-TZVP level based on optimized ground-state geometries.

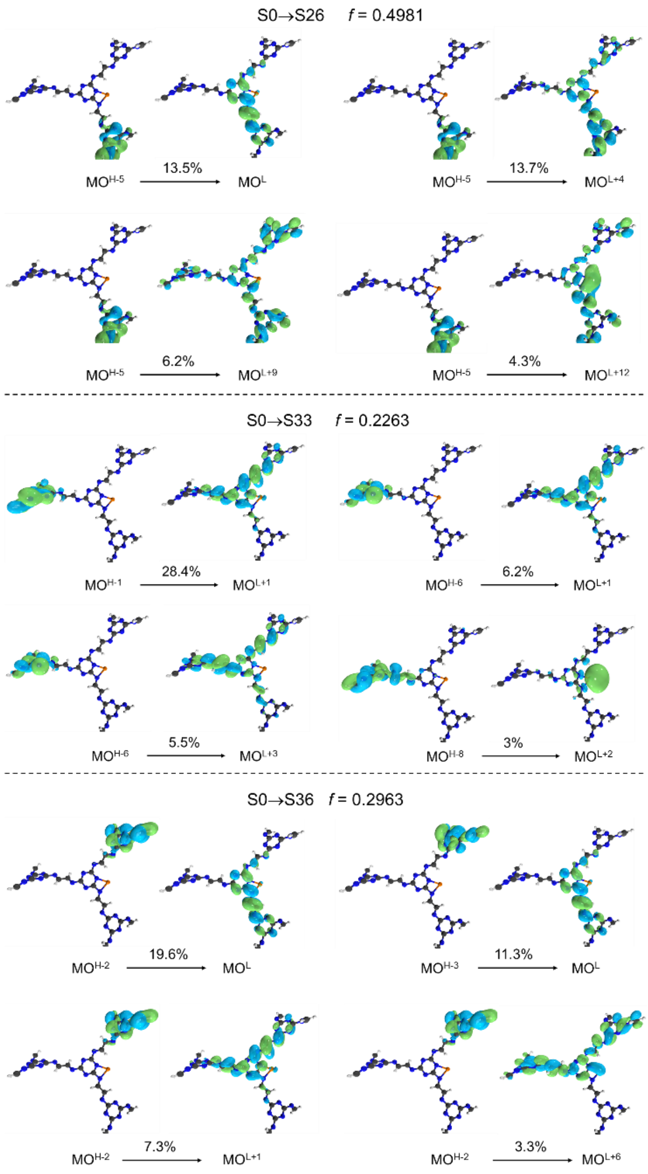

Supplementary Fig. 34 Molecular orbital transitions for the three highest intensity of delocalization excitations in $Cu_{SA}C_6N_6$: S0→S26, S0→S33, S0→S36. The corresponding oscillator strengths (*f*) was marked after the excitation. Isosurfaces (isovalue = 0.02 au) of the HOMO-n (left, n⩾0) and LUMO+n (right, n≥0), green and blue regions denote the positive and negative orbital phases, respectively. The numbers in the middle of the arrow denote the contributions of the transition to the corresponding excitation. Carbon atoms (black), Nitrogen atoms (blue), Hydrogen atoms (white), Cu atoms (orange). Electron excitations were calculated with M06-2X/def2-TZVP level based on optimized ground-state geometries.

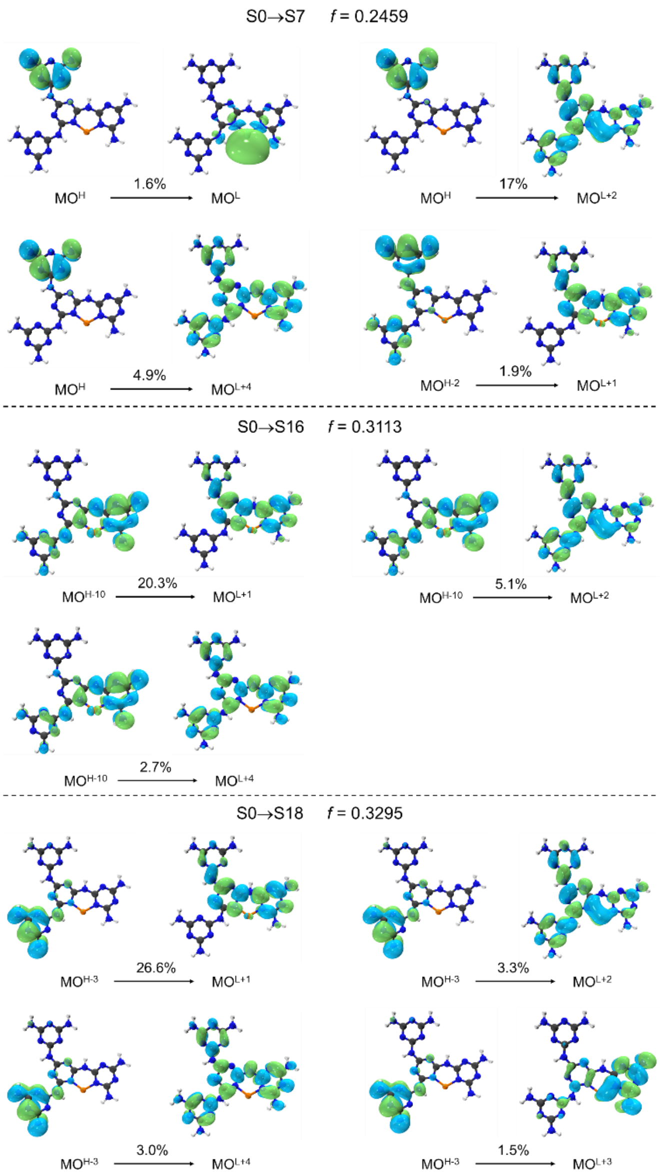

Supplementary Fig. 35 Molecular orbital transitions for the three highest intensity of delocalization excitations in Cu-g-C_3N_4: S0→S7, S0→S16, S0→S18. The corresponding oscillator strengths (*f*) was marked after the excitation. Isosurfaces (isovalue = 0.02 au) of the HOMO-n (left, n⩾0) and LUMO+n (right, n≥0), green and blue regions denote the positive and negative orbital phases, respectively. The numbers in the middle of the arrow denote the contributions of the transition to the corresponding excitation. Carbon atoms (black), Nitrogen atoms (blue), Hydrogen atoms (white), Cu atoms (orange). Electron excitations were calculated with M06-2X/def2-TZVP level based on optimized ground-state geometries.

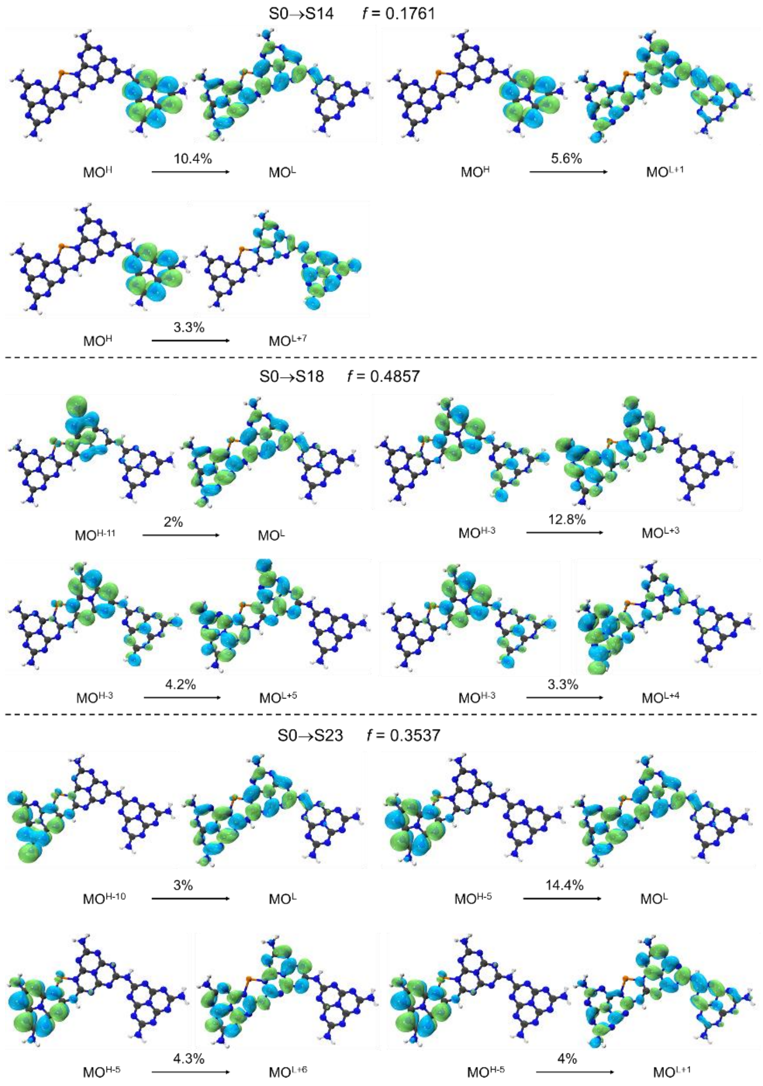

Supplementary Fig. 36 Molecular orbital transitions for the three highest intensity of delocalization excitations in Cu-PCN: S0→S14, S0→S18, S0→S23. The corresponding oscillator strengths (*f*) was marked after the excitation. Isosurfaces (isovalue = 0.02 au) of the HOMO-n (left, n⩾0) and LUMO+n (right, n≥0), green and blue regions denote the positive and negative orbital phases, respectively. The numbers in the middle of the arrow denote the contributions of the transition to the corresponding excitation. Carbon atoms (black), Nitrogen atoms (blue), Hydrogen atoms (white), Cu atoms (orange). Electron excitations were calculated with M06-2X/def2-TZVP level based on optimized ground-state geometries.

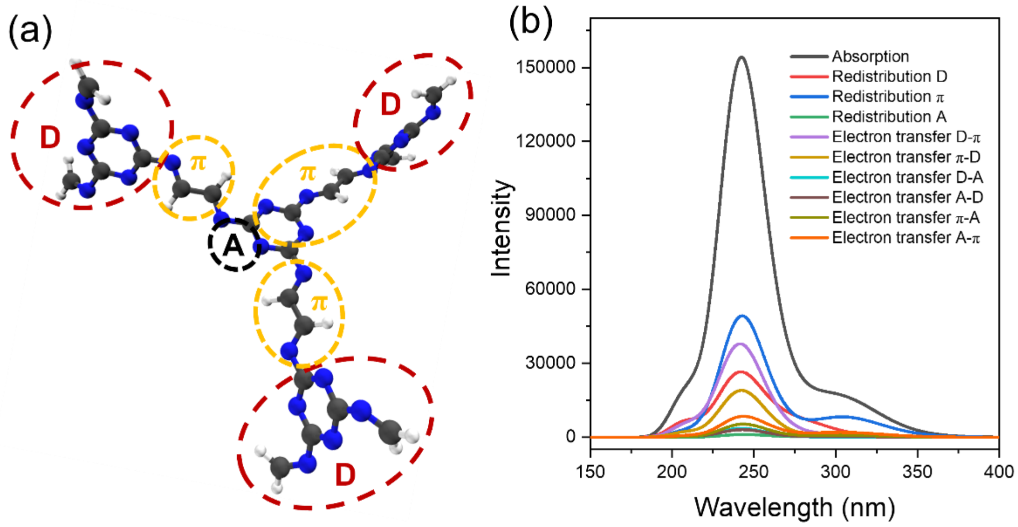

Supplementary Fig. 37 Partition and CTS of C_6N_6. (a) Ball-and-stick model structures of C_6N_6. Dashed areas indicate donor (D), π-conjugated charge transfer channels (π), and acceptor (A). Carbon atoms (black), Nitrogen atoms (blue), Hydrogen atoms (white), Cu atoms (orange). (b) Simulated absorption spectrum and CTS of C_6N_6. Electron excitations were calculated with M06-2X/def2-TZVP level based on optimized ground-state geometries.

For a better scholarly presentation, the structure of $Cu_{SA}C_6N_6$ and the control C_6N_6 have been additionally calculated. As shown in **Fig. 5b** and **Supplementary Fig. 37a**, owing to the acceptor was changed from a =N-Cu-N= to two N atoms, the contribution of D-A in the CTS (**Fig. 5c** and **Supplementary Fig. 37b**) decreased from 9.6% ($Cu_{SA}C_6N_6$) to 2.2% (C_6N_6). It was worth noting that the existence of Cu atom in $Cu_{SA}C_6N_6$ broke the symmetry, demonstrating that $Cu_{SA}C_6N_6$ accepted electrons more readily than C_6N_6 that were excited from three triazine rings (donor).

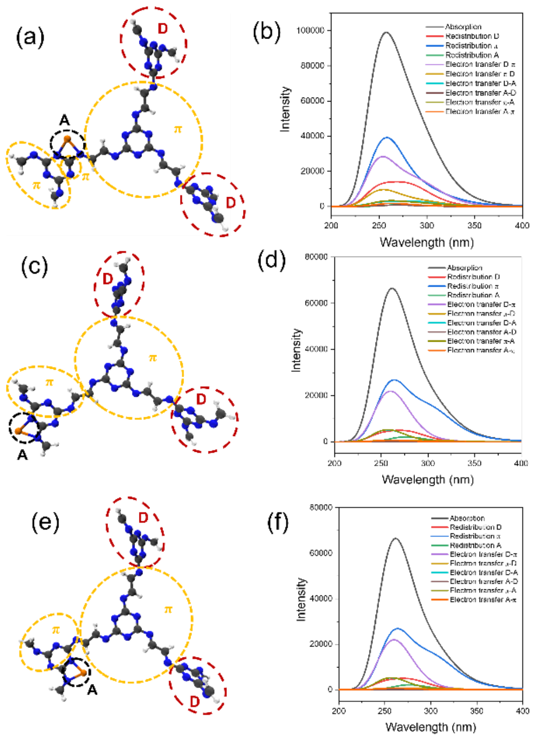

Supplementary Fig. 38 Partition and CTS of $Cu_{SA}C_6N_6$ with different metal-anchoring positions. (a, c, e) Ball-and-stick model structures of $Cu_{SA}C_6N_6$ with three possible metal-anchoring positions. Dashed areas indicate donor (D), π-conjugated charge transfer channels (π), and acceptor (A). Carbon atoms (black), Nitrogen atoms (blue), Hydrogen atoms (white), Cu atoms (orange). (b, d, f) Simulated absorption spectrum and CTS of $Cu_{SA}C_6N_6$. Electron excitations were calculated with M06-2X/def2-TZVP level based on optimized ground-state geometries.

To comprehensive analysis of the computational description for delocalization, the CTS of $Cu_{SA}C_6N_6$ with four possible metal-anchoring positions were calculated. In these structures, Cu was anchored to two N atoms at the central/edge triazine ring. As shown in **Fig. 5b, Supplementary Fig. 38b**, **38d**, and **38f,** the contribution of D-A transfer transition in the CTS were 9.6%, 3.8%, 6.3%, and 7.6%, respectively, much higher than that without Cu anchored (**Supplementary Fig. 37b**, 2.2%), indicative of an increased delocalization by the Cu atom.

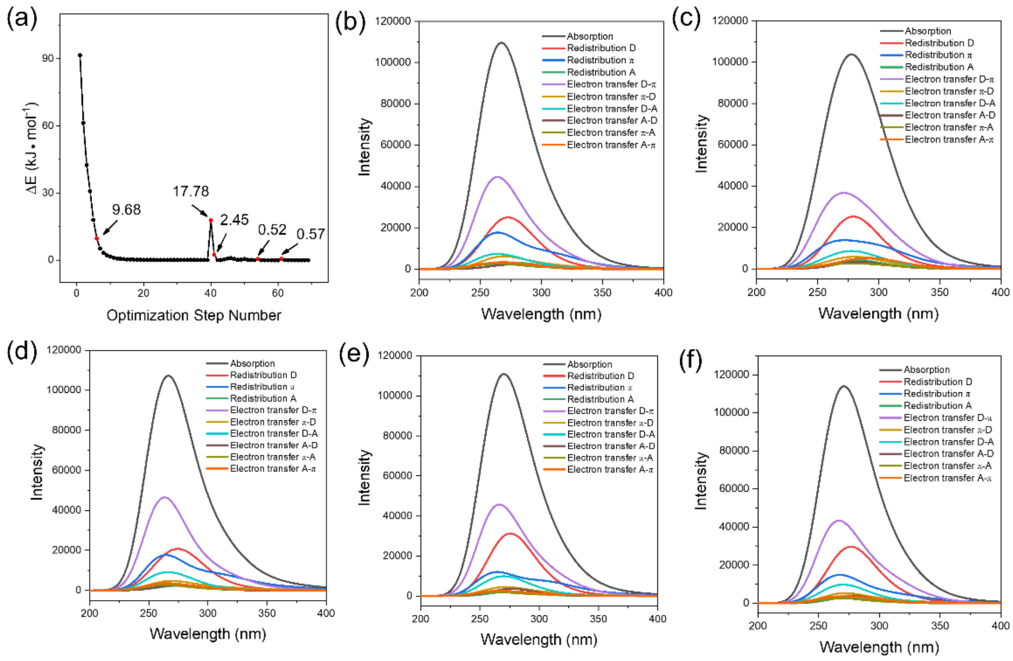

Supplementary Fig. 39 Energy curve of optimization and corresponding CTS of $Cu_{SA}C_6N_6$ structures. (a) Energy curve of $Cu_{SA}C_6N_6$ optimized from the initial guess to the point of minimal energy. The five points marked in red correspond to the structure of the calculated CTS shown in (b-f) from left to right, in that order. Electron excitations were calculated with M06-2X/def2-TZVP level based on optimized ground-state geometries.

To comprehensive analysis of the computational description for conformational flexibility, five typical structures were randomly extracted from the optimization process (**Supplementary Fig. 39a**). The energy difference between these structures and the energy minimum structure was less than 18 kJ/mol, corresponding to the requirement of rotation energy for the C-C bond in ethane. It was aimed to simulate the different structures arisen from the thermal motion of the molecule. As shown in the CTS of $Cu_{SA}C_6N_6$ (**Supplementary Fig. 39b-S39f**), the contribution of D-A transfer transition ranged from 7.1 to 9.0%, close to that of the energy minimum structure (**Fig 5c**, 9.6%). These results indicated that conformational flexibility almost had no side effect on the excited state via the D-π-A electron-transfer.

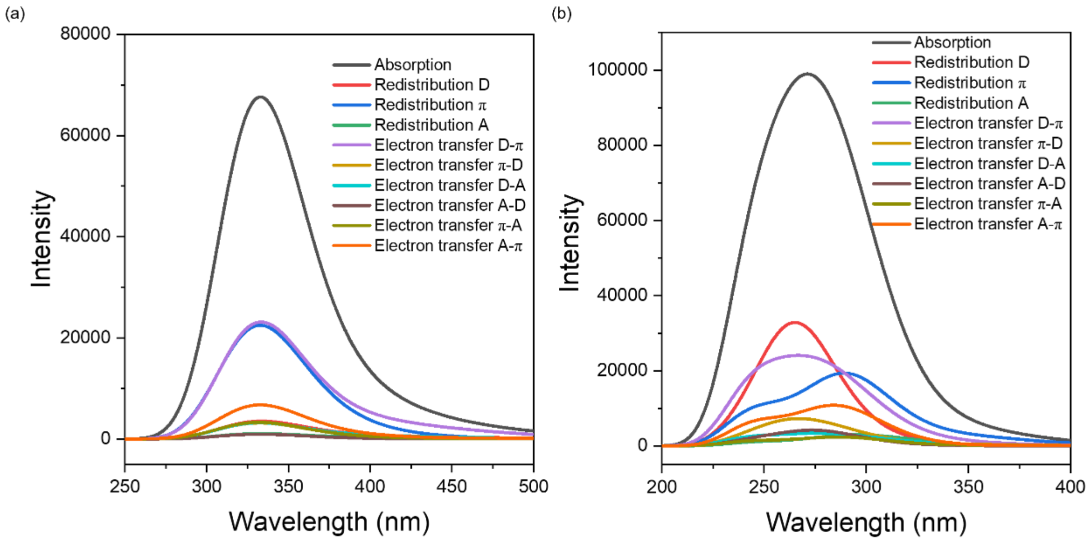

Supplementary Fig. 40 Simulated absorption spectrum and CTS of $Cu_{SA}C_6N_6$. Electron excitations were calculated with (a) PBE0/def2-TZVP and (b) ωB97XD/def2-TZVP based on optimized ground-state geometries.

For the singlet excited state, the composition of the Hartree-Fock function in the hybrid functional has a systematic influence on the results. To comprehensive analysis of the effect of the hybrid functionals, as control, M06-2X (54% Hartree-Fock function),[7] PBE0 (25% Hartree-Fock function)[9,10] and ωB97XD (22.2% Hartree-Fock function)[11,12] have been chosen for comparison. As shown in **Supplementary Fig. 40**, the CTS calculated by PBE0 and ωB97XD also proved that the excitation of the charge separation mainly consisted of D-π and D-A.

Supplementary Table 1 Combustion elemental analysis of $Cu_{SA}C_6N_6$ and Cu-$CN_{int.}$

sample	C [wt.%]	N [wt.%]	H [wt.%]	C/N [molar ratio]
$Cu_{SA}C_6N_6$	39.63	48.46	2.15	0.95
Cu-$CN_{int.}$	31.24	50.07	4.6	0.73

Supplementary Table 2 EXAFS fitting parameters at the Cu K-edge for various samples. (S_0^2=0.818)

Sample	1st shell	CN	R(Å)	σ^2	ΔE_0（eV）	R factor
Cu foil	Cu-Cu	12	2.52	0.0083	3.1	0.0070
$Cu_{SA}C_6N_6$	Cu-N	2	1.93	0.0060	-4.8	0.043

$^a N$: coordination numbers; $^b R$: bond distance; $^c \sigma^2$: Debye-Waller factors; $^d \Delta E_0$: the inner potential correction. *R* factor: goodness of fit.

Kinetic equation of glucose sensor under light irradiation of different intensity.

The intelligent response of glucose in basic reaction is indeed a typical kinetics curve for enzymatic reactions, and the involved reactions can be described follows.[19]

$$[GO_x] + [GLu] \underset{k_{-1}}{\overset{k_1}{\rightleftharpoons}} [GO_x\ GLu] \tag{S1}$$

$$[GO_x\ GLu] \overset{k_2}{\rightarrow} [GO_x] + [H_2O_2] + [Gluconic\ acid\ ] \tag{S2}$$

$$[Cu_{SA}C_6N_6] + [H_2O_2] \underset{k_{-3}}{\overset{k_3}{\rightleftharpoons}} [Cu_{SA}C_6N_6\ H_2O_2] \tag{S3}$$

$$[Cu_{SA}C_6N_6\ H_2O_2] + [ABTS] \underset{k_{-4}}{\overset{k_4}{\rightleftharpoons}} [ABTS\ Cu_{SA}C_6N_6\ H_2O_2] \tag{S4}$$

$$[ABTS\ Cu_{SA}C_6N_6\ H_2O_2] \overset{k_5}{\rightarrow} ABTS_{ox} + Cu_{SA}C_6N_6 + H_2O \tag{S5}$$

where the intermediate species (except for H_2O_2) is described in brackets.

As the overall reaction rate would be calculated by the formation rate of the $ABTS_{ox}$ product, according to the law of mass action, the reaction rate equations are:

$$v = \frac{d[ABTS_{ox}]}{dt} = k_5[ABTS\ Cu_{SA}C_6N_6\ H_2O_2] \tag{S6}$$

where the v is reaction rate of $[ABTS_{ox}]$.

Here, $[Cu_{SA}C_6N_6\ H_2O_2]$ and $[ABTS\ Cu_{SA}C_6N_6\ H_2O_2]$ are approximately considered to be stable state, thus according to the stationary state approximation method,

$$\frac{d[ABTS\ Cu_{SA}C_6N_6\ H_2O_2]}{dt} = k_4[Cu_{SA}C_6N_6\ H_2O_2][ABTS] - k_{-4}[ABTS\ Cu_{SA}C_6N_6\ H_2O_2] - k_5[ABTS\ Cu_{SA}C_6N_6\ H_2O_2] = 0 \tag{S7}$$

$$\frac{d[Cu_{SA}C_6N_6\ H_2O_2]}{dt} = k_3[Cu_{SA}C_6N_6][H_2O_2] - k_{-3}[Cu_{SA}C_6N_6\ H_2O_2] - k_4[Cu_{SA}C_6N_6\ H_2O_2][ABTS] + k_{-4}[ABTS\ Cu_{SA}C_6N_6\ H_2O_2] = 0 \tag{S8}$$

Then, the $[ABTS\ Cu_{SA}C_6N_6\ H_2O_2]$ can be represented by **Eq. 9**,

$$[Cu_{SA}C_6N_6\ H_2O_2] = \frac{(k_{-4}+k_5)[ABTS\ Cu_{SA}C_6N_6\ H_2O_2]}{k_4[ABTS]} \tag{S9}$$

Next, add the **Eq. S7** and **Eq. S8**,

$$k_3[Cu_{SA}C_6N_6][H_2O_2] - k_{-3}[Cu_{SA}C_6N_6\ H_2O_2] - k_5[ABTS\ Cu_{SA}C_6N_6\ H_2O_2] = 0 \quad \text{(S10)}$$

For **Eq. S10**, by substituting [ABTS $Cu_{SA}C_6N_6$ H_2O_2] with **Eq. S9**, it can be written as:

$$k_3[Cu_{SA}C_6N_6][H_2O_2] - \frac{k_3(k_{-4}+k_5)[ABTS\ Cu_{SA}C_6N_6\ H_2O_2]}{k_4[ABTS]} - k_5[ABTS\ Cu_{SA}C_6N_6\ H_2O_2] = 0$$

(S11)

Here, given that [$Cu_{SA}C_6N_6$] = 1 and according to the stationary state approximation method, [ABTS $Cu_{SA}C_6N_6$ H_2O_2] can be represented by **Eq. S12**,

$$[ABTS\ Cu_{SA}C_6N_6\ H_2O_2] = \frac{k_3k_4[ABTS][H_2O_2]}{k_{-3}k_{-4}+k_{-3}k_5+k_4k_5[ABTS]} \quad \text{(S12)}$$

Substituting [ABTS $Cu_{SA}C_6N_6$ H_2O_2] from **Eq. S12** into **Eq. S6**, we can obtain

$$v = \frac{d[ABTS_{ox}]}{dt} = \frac{k_5k_3k_4[ABTS][H_2O_2]}{k_{-3}k_{-4}+k_{-3}k_5+k_4k_5[ABTS]} \quad \text{(S13)}$$

For the **Eq. S2**

$$v = \frac{d[H_2O_2]}{dt} = k_2[Glu\ GO_x] \quad \text{(S14)}$$

Here, given that [GO_X] = 1 and according to the equilibrium state approximation method, we can obtain,

$$\frac{d[Glu\ GO_x]}{dt} = k_1[Glu][GO_x] - k_{-1}[Glu\ GO_x] - k_2[Glu\ GO_x] = 0 \quad \text{(S15)}$$

$$[Glu\ GO_x] = \frac{k_1[Glu]}{k_{-1}+k_2} \quad \text{(S16)}$$

Then, based on **Eq. S15** and **Eq. S16**, **Eq. S14** can be written as:

$$\frac{d[H_2O_2]}{dt} = \frac{k_1k_2[Glu]}{k_{-1}+k_2} \quad \text{(S17)}$$

Taking the indefinite integral for **Eq. S17**, we can obtain:

$$\int_0^{t_1} d[H_2O_2] = \int_0^{t_1} \frac{k_1k_2[Glu]}{k_{-1}+k_2} dt_1 \quad \text{(S18)}$$

$$[H_2O_2] = \frac{k_1k_2[Glu]t_1}{k_{-1}+k_2} + a \quad \text{(S19)}$$

where a is a constant.

Substituting [H_2O_2] with **Eq. S19**, **Eq. S13** can be written as:

$$\frac{d[ABTS_{ox}]}{dt} = \frac{k_1k_2k_5k_3k_4[ABTS][Glu]t_1+(k_{-1}+k_2)k_5k_3k_4[ABTS]a}{(k_{-1}+k_2)(k_{-3}k_{-4}+k_{-3}k_5+k_4k_5[ABTS])} \qquad (S20)$$

Taking the definite integral for **Eq. S20**, we can obtain:

$$\int_0^{t_2} d[ABTS_{ox}] = \int_0^{t_2} \frac{k_1k_2k_5k_3k_4[ABTS][Glu]t_1+(k_{-1}+k_2)k_5k_3k_4[ABTS]a}{(k_{-1}+k_2)(k_{-3}k_{-4}+k_{-3}k_5+k_4k_5[ABTS])} dt_2 \qquad (S21)$$

When $t_2 = 0$, the initial concentration of ABTS is 0, i.e., $[ABTS_{ox}]_0 = 0$,

$$[ABTS_{ox}]_{t_2} = \frac{k_1k_2k_5k_3k_4[ABTS][Glu]t_1t_2+(k_{-1}+k_2)k_5k_3k_4[ABTS]at_1}{(k_{-1}+k_2)(k_{-3}k_{-4}+k_{-3}k_5+k_4k_5[ABTS])} + b \qquad (S22)$$

Generally, t_1(time for glucose oxidation), t_2 (time for ABTS oxidation), a and b are constant in experiments, **Eq. S22** can be written as:

$$[ABTS_{ox}] = k_B[Glu] + c_1 \qquad (S23)$$

where the k_B is the slope of the basic reaction, it can be written as:

$$k_B = \frac{k_1k_2k_5k_3k_4[ABTS]t_1t_2}{(k_{-1}+k_2)(k_{-3}k_{-4}+k_{-3}k_5+k_4k_5[ABTS])} \qquad (S24)$$

And the intercept:

$$c_1 = \frac{(k_{-1}+k_2)k_5k_3k_4[ABTS]at_1}{(k_{-1}+k_2)(k_{-3}k_{-4}+k_{-3}k_5+k_4k_5[ABTS])} + b \qquad (S25)$$

where c_1 is a constant.

According to the Lambert-Beer law, [$ABTS_{ox}$] is proportional to its absorbance at 417 nm. Therefore, **Eq. S23** well explains the linear relationship between the absorbance of $ABTS_{ox}$ at 417 nm and [Glu] (**Fig. 6b**).

Accordingly, the intelligent response of glucose in gain reaction under light irradiation of different intensity, the involved reactions can be described as follows:

$$[GO_x] + [GLu] \underset{k_{-1}}{\overset{k_1}{\rightleftharpoons}} [GO_x\ GLu] \tag{S1}$$

$$[GO_x\ GLu] \overset{k_2}{\rightarrow} [GO_x] + [H_2O_2] + [Gluconic\ acid\ ] \tag{S2}$$

$$[Cu_{SA}^{+}C_6N_6] + hv \overset{k_3}{\rightarrow} [Cu_{SA}^{+}C_6N_6^{*}] \tag{S3}$$

$$[Cu_{SA}^{+}C_6N_6^{*}] \overset{k_4}{\rightarrow} [Cu_{SA}^{0}C_6N_6] \tag{S4}$$

$$[Cu_{SA}^{+}C_6N_6^{*}] + [Cu_{SA}^{+}C_6N_6] \overset{k_5}{\rightarrow} 2[Cu_{SA}^{+}C_6N_6] \tag{S5}$$

$$[Cu_{SA}^{0}C_6N_6] + [H_2O_2] \underset{k_{-6}}{\overset{k_6}{\rightleftharpoons}} [Cu_{SA}^{0}C_6N_6\ H_2O_2] \tag{S6}$$

$$[Cu_{SA}^{0}C_6N_6\ H_2O_2] + [ABTS] \underset{k_{-7}}{\overset{k_7}{\rightleftharpoons}} [ABTS\ Cu_{SA}^{0}C_6N_6\ H_2O_2] \tag{S7}$$

$$[ABTS\ Cu_{SA}^{0}C_6N_6\ H_2O_2] \overset{k_8}{\rightarrow} ABTS_{ox} + Cu_{SA}^{+}C_6N_6 + H_2O \tag{S8}$$

Here, $[Cu^{+}_{SA}C_6N_6^{*}]$ is approximately considered to be stable state in light irradiation process, thus according to the stationary state approximation method,

$$\frac{d[Cu_{SA}^{+}C_6N_6^{*}]}{dt} = k_3 I_a - k_4[Cu_{SA}^{+}C_6N_6^{*}] - k_5[Cu_{SA}^{+}C_6N_6^{*}][Cu_{SA}^{+}C_6N_6] = 0 \tag{S9}$$

$$[Cu_{SA}^{+}C_6N_6^{*}] = \frac{k_3 I_a}{k_4 + k_5[Cu_{SA}^{+}C_6N_6]} \tag{S10}$$

Thus, the reaction rate of $[Cu^{0}_{SA}C_6N_6]$ can be written as **Eq. S11**,

$$v = \frac{d[Cu_{SA}^{0}C_6N_6]}{dt} = k_4[Cu_{SA}^{+}C_6N_6^{*}] \tag{S11}$$

Substituting $[Cu^{+}_{SA}C_6N_6^{*}]$ with **Eq. S10**, the **Eq. S11** can be written as,

$$\frac{d[Cu_{SA}^{0}C_6N_6]}{dt} = \frac{k_4 k_3 I_a}{k_4 + k_5[Cu_{SA}^{+}C_6N_6]} \tag{S12}$$

Taking the indefinite integral for **Eq. S12**, we can obtain:

$$\int_0^{t_2} d[Cu_{SA}^{0}C_6N_6] = \int_0^{t_2} \frac{k_4 k_3 I_a}{k_4 + k_5[Cu_{SA}^{+}C_6N_6]} dt_2 \tag{S13}$$

$$[Cu_{SA}^{0}C_6N_6] = \frac{k_4 k_3 I_a t_2}{k_4 + k_5[Cu_{SA}^{+}C_6N_6]} + d \tag{S14}$$

where d is a constant.

Next, as the overall reaction rate would be calculated by the formation rate of the $ABTS_{ox}$ product, according to the law of mass action, the reaction rate equations are:

$$v = \frac{d[ABTS_{ox}]}{dt} = k_8[ABTS\ Cu_{SA}^0 C_6 N_6\ H_2O_2] \quad (S15)$$

Here, $[Cu^0{}_{SA}C_6N_6\ H_2O_2]$ and $[ABTS\ Cu^0{}_{SA}C_6N_6\ H_2O_2\ H_2O_2]$ are approximately considered to be stable state, thus according to the stationary state approximation method, $[ABTS\ Cu^0{}_{SA}C_6N_6\ H_2O_2]$ can be represented by **Eq. S16**,

$$[Cu_{SA}^0 C_6 N_6\ H_2O_2] = \frac{(k_{-7}+k_8)[ABTS\ Cu_{SA}^0 C_6 N_6\ H_2O_2]}{k_7[ABTS]} \quad (S16)$$

Given that $[Cu_{SA}C_6N_6] = 1$, $[ABTS\ Cu_{SA}C_6N_6\ H_2O_2]$ can be represented by **Eq. S17**,

$$[ABTS\ Cu_{SA}^0 C_6 N_6\ H_2O_2] = \frac{k_4k_3I_{\mathrm{a}}t_2k_6k_7[ABTS][H_2O_2]+(k_3+k_4)dk_5k_6[ABTS]}{(k_{-6}k_{-7}+k_{-6}k_8+k_7k_8[ABTS])(k_4+k_5)} \quad (S17)$$

Substituting $[ABTS\ Cu^0{}_{SA}C_6N_6\ H_2O_2]$ with **Eq. S17**, the **Eq. S15** can be written as

$$v = \frac{d[ABTS_{ox}]}{dt} = \frac{k_4k_3I_{\mathrm{a}}t_2k_6k_7k_8[ABTS][H_2O_2]+(k_4+k_5)dk_6k_7k_8[ABTS]}{(k_{-6}k_{-7}+k_{-6}k_8+k_7k_8[ABTS])(k_4+k_5)} \quad (S18)$$

where $[H_2O_2]$ was calculated from above.

$$[H_2O_2] = \frac{k_1k_2[Glu]t_1}{k_{-1}+k_2} + a \quad (S19)$$

Substituting $[H_2O_2]$ with **Eq. S19**, **Eq. S18** can be written as:

$$\frac{d[ABTS_{ox}]}{dt} = \frac{k_4k_3I_{\mathrm{a}}t_2t_1k_1k_2k_6k_7k_8[ABTS][Glu]+((k_4k_3I_{\mathrm{a}}t_1a(k_{-1}+k_2)+d(k_4+k_5))k_6k_7k_8[ABTS]}{(k_{-1}+k_2)(k_4+k_5)(k_{-6}k_{-7}+k_{-6}k_8+k_7k_8[ABTS])} \quad (S20)$$

Similarly,

$$[ABTS_{ox}]_{t_2} = \frac{k_4k_3I_{\mathrm{a}}t_2^2t_1k_1k_2k_6k_7k_8[ABTS][Glu]+((k_4k_3I_{\mathrm{a}}t_1a(k_{-1}+k_2)+d(k_4+k_5))k_6k_7k_8[ABTS]}{(k_{-1}+k_2)(k_4+k_5)(k_{-6}k_{-7}+k_{-6}k_8+k_7k_8[ABTS])} + e$$

(S21)

Generally, t_1 (time for glucose oxidation), t_2 (time for ABTS oxidation), a, d and e are constants in experiments, and **Eq. S21** can be written as:

$$[ABTS_{ox}] = k_G[Glu] + f \quad (S22)$$

where f is a constant.

Here, the k_G is the slope of gain reaction which increases with the enhancement of I_a.

It can be written as:

$$k_G = \frac{I_a k_4 k_3 k_1 k_2 k_6 k_7 k_8 [ABTS] t_1 t_2^2}{(k_{-1}+k_2)(k_4+k_5)(k_{-6}k_{-7}+k_{-6}k_8+k_7k_8[ABTS])} \quad \text{(S23)}$$

We can see that k_G is proportional to I_a.

And the intercept:

$$f = \frac{((k_4 k_3 I_a t_1 a(k_{-1}+k_2)+d(k_4+k_5))k_6 k_7 k_8 [ABTS]}{(k_{-1}+k_2)(k_4+k_5)(k_{-6}k_{-7}+k_{-6}k_8+k_7k_8[ABTS])} + e \quad \text{(S24)}$$

Finally, the total [$ABTS_{ox}$] is addition of basic reaction and gain reaction.

$$[ABTS_{ox}] = (k_G + k_B)[Glu] + c_2 \quad \text{(S25)}$$

where c_2 is a constant.

Here, under light irradiation of different intensity, **Eq. S25** could well explain the light intensity-dependent linear detection range and sensitivity to a diverse range of concentrations in vitro (**Fig. 6b**).

References

1 Zhou, Z. *et al.* Chemically Modulated Carbon Nitride Nanosheets for Highly Selective Electrochemiluminescent Detection of Multiple Metal-ions. *Anal. Chem.* **88**, 6004-6010 (2016).

2 Ju, E. *et al.* Copper(II)-Graphitic Carbon Nitride Triggered Synergy: Improved ROS Generation and Reduced Glutathione Levels for Enhanced Photodynamic Therapy. *Angew. Chem. Int. Ed.* **55**, 11467-11471 (2016).

3 Wu, Y. *et al.* Cascade Reaction System Integrating Single-Atom Nanozymes with Abundant Cu Sites for Enhanced Biosensing. *Anal. Chem.* **92**, 3373-3379 (2020).

4 Huang, C. *et al.* Unraveling fundamental active units in carbon nitride for photocatalytic oxidation reactions. *Nat. Commun.* **12**, 320 (2021).

5 Wei, H. & Wang, E. Fe_3O_4 Magnetic Nanoparticles as Peroxidase Mimetics and Their Applications in H_2O_2 and Glucose Detection. *Anal. Chem.* **80**, 2250-2254 (2008).

6 Gaussian 16 Rev. C.02 (Wallingford, CT, 2016).

7 Jacquemin, D., Planchat, A., Adamo, C. & Mennucci, B. TD-DFT Assessment of Functionals for Optical 0–0 Transitions in Solvated Dyes. *J. Chem. Theory Comput.* **8**, 2359-2372 (2012).

8 Weigend, F. & Ahlrichs, R. Balanced basis sets of split valence, triple zeta valence and quadruple zeta valence quality for H to Rn: Design and assessment of accuracy. *Phys. Chem. Chem. Phys.* **7**, 3297-3305 (2005).

9 Jacquemin, D., Mennucci, B. & Adamo, C. Excited-state calculations with TD-DFT: from benchmarks to simulations in complex environments. *Phys. Chem. Chem. Phys.* **13** (2011).

10 Dahlke, E. E. & Truhlar, D. G. Electrostatically Embedded Many-Body Expansion for Simulations. *J. Chem. Theory Comput.* **4**, 1-6 (2007).

11 Jacquemin, D., Brémond, E., Planchat, A., Ciofini, I. & Adamo, C. TD-DFT Vibronic Couplings in Anthraquinones: From Basis Set and Functional

Benchmarks to Applications for Industrial Dyes. *J. Chem. Theory Comput.* **7**, 1882-1892 (2011).

12 Shi, B., Yuan, L., Tang, T., Yuan, Y. & Tang, Y. Study on electronic structure and excitation characteristics of cyclo[18]carbon. *Chem. Phys. Lett.* **741**, 136975-136981 (2020).

13 Liu, Z., Lu, T. & Chen, Q. An sp-hybridized all-carboatomic ring, cyclo[18]carbon: Electronic structure, electronic spectrum, and optical nonlinearity. *Carbon* **165**, 461-467 (2020).

14 Lu, T. & Chen, F. Multiwfn: A multifunctional wavefunction analyzer. *J. Comput. Chem.* **33**, 580-592 (2012).

15 Liu, Z., Wang, X., Lu, T., Yuan, A. & Yan, X. Potential optical molecular switch: Lithium@cyclo[18]carbon complex transforming between two stable configurations. *Carbon* **187**, 78-85 (2022).

16 Duan, Y. *et al.* Optically Active Chiral CuO "Nanoflowers". *J. Am. Chem. Soc.* **136**, 7193-7196 (2014).

17 Liu, Y. *et al.* In vitro measurement of superoxide dismutase-like nanozyme activity: a comparative study. *Analyst* **146**, 1872-1879 (2021).

18 Nosaka, Y. & Nosaka, A. Y. Generation and Detection of Reactive Oxygen Species in Photocatalysis. *Chem. Rev.* **117**, 11302-11336 (2017).

19 Zhou, Q. *et al.* Cascaded Nanozyme System with High Reaction Selectivity by Substrate Screening and Channeling in a Microfluidic Device. *Angew. Chem. Int. Ed.* **61**, e202112453 (2021).
